# The human cardiac and skeletal muscle proteomes defined by transcriptomics and antibody-based profiling

**DOI:** 10.1186/s12864-015-1686-y

**Published:** 2015-06-25

**Authors:** Cecilia Lindskog, Jerker Linné, Linn Fagerberg, Björn M Hallström, Carl Johan Sundberg, Malene Lindholm, Mikael Huss, Caroline Kampf, Howard Choi, David A Liem, Peipei Ping, Leif Väremo, Adil Mardinoglu, Jens Nielsen, Erik Larsson, Fredrik Pontén, Mathias Uhlén

**Affiliations:** Science for Life Laboratory, Dept of Immunology Genetics and Pathology, Uppsala University, SE-751 85 Uppsala, Sweden; Science for Life Laboratory, KTH - Royal Institute of Technology, AlbaNova University Center, SE-171 21 Stockholm, Sweden; Department of Physiology and Pharmacology, Karolinska Institutet, SE-171 77 Stockholm, Sweden; Science for Life Laboratory, Dept of Biochemistry and Biophysics, Stockholm University, Box 1031, SE-17121 Solna, Sweden; NHLBI Proteomics Center at UCLA, Departments of Physiology and Medicine, Division of Cardiology, David Geffen School of Medicine, University of California, Los Angeles, CA USA; Department of Chemical and Biological Engineering, Chalmers University of Technology, SE-412 58 Gothenburg, Sweden; Department of Immunology, Genetics and Pathology, Rudbeck Laboratory, Uppsala University, SE-751 85 Uppsala, Sweden

**Keywords:** Transcriptome, Proteome, Cardiac and skeletal muscle

## Abstract

**Background:**

To understand cardiac and skeletal muscle function, it is important to define and explore their molecular constituents and also to identify similarities and differences in the gene expression in these two different striated muscle tissues. Here, we have investigated the genes and proteins with elevated expression in cardiac and skeletal muscle in relation to all other major human tissues and organs using a global transcriptomics analysis complemented with antibody-based profiling to localize the corresponding proteins on a single cell level.

**Results:**

Our study identified a comprehensive list of genes expressed in cardiac and skeletal muscle. The genes with elevated expression were further stratified according to their global expression pattern across the human body as well as their precise localization in the muscle tissues. The functions of the proteins encoded by the elevated genes are well in line with the physiological functions of cardiac and skeletal muscle, such as contraction, ion transport, regulation of membrane potential and actomyosin structure organization. A large fraction of the transcripts in both cardiac and skeletal muscle correspond to mitochondrial proteins involved in energy metabolism, which demonstrates the extreme specialization of these muscle tissues to provide energy for contraction.

**Conclusions:**

Our results provide a comprehensive list of genes and proteins elevated in striated muscles. A number of proteins not previously characterized in cardiac and skeletal muscle were identified and localized to specific cellular subcompartments. These proteins represent an interesting starting point for further functional analysis of their role in muscle biology and disease.

**Electronic supplementary material:**

The online version of this article (doi:10.1186/s12864-015-1686-y) contains supplementary material, which is available to authorized users.

## Background

Cardiac and skeletal muscles are both striated muscles composed of repeated units called sarcomeres, crossed with a regular pattern of fine red and white lines giving the muscles their distinctive striated appearance and their name. The cells are rich in mitochondria and contain to a large extent myosin and actin proteins, signifying different regions of the sarcomeres. While skeletal muscle consists of parallel linear fibers, the cardiac muscle cells (cardiomyocytes) are arranged in fibers exhibiting cross-striations formed by alternating segments of thick and thin protein filaments. For rhythmical and synchronized contraction, cardiomyocytes form a functional syncytium via intercalated discs, where the membranes of adjacent cells are situated closely together. Intercalated discs are unique for the heart and visible as clearly distinguishable bands between cardiomyocytes. In addition to muscle fibers and vasculature, the heart also includes streaks of connective tissue and cuffs of adipose tissue surrounding the smaller vessels. Skeletal muscles consist of large multinucleated myocytes called syncytia, formed during development via fusion of myotubes. There are two main muscle fiber types (slow type I and fast type II) depending on the dominant type of myosin present. Skeletal muscles are attached to bone and contract voluntarily (via nerve stimulation) as opposed to cardiac muscle and smooth muscle. In addition to the muscle fibers, skeletal muscle samples also contain streaks of adipose tissue and connective tissue with fibroblasts. Skeletal muscle is highly vascularized with a fine network of capillary endothelial cells running in between the fibers.

Detailed knowledge of the molecular constituents of striated muscles under normal conditions is essential to understand the cellular interactions in health and disease. Thus far, several transcriptomics signatures on diseased hearts have been performed, primarily using animal models or human serum [1–3]. These studies have provided important insights into disease pathogenesis and drug development, however, until date, no detailed characterization of the transcriptome and proteome in normal human striated muscle tissues has been performed using a genome-wide analysis of both the transcripts and the corresponding protein profiles. In addition, no comprehensive study has yet been performed to compare the cardiac and skeletal muscle to identify both unique and shared proteins involved in muscle biology.

Here, we generated next generation sequencing (RNA-seq) data for skeletal muscle tissue and performed an extended analysis based on the RNA-seq data of 28 different tissues involving 100 samples, including four and five individual samples from cardiac and skeletal muscle, respectively. This approach has been used for identifying a comprehensive list of several hundred genes elevated in striated muscles as compared to other analyzed tissues, representing all major organs in the human body. Subsequently, an antibody-based approach using tissue microarrays and immunohistochemistry was used to localize the elevated proteins to different subcompartments of heart and skeletal muscle on a single cell level.

## Results

### The transcriptomic analysis

**Fig. 1:**
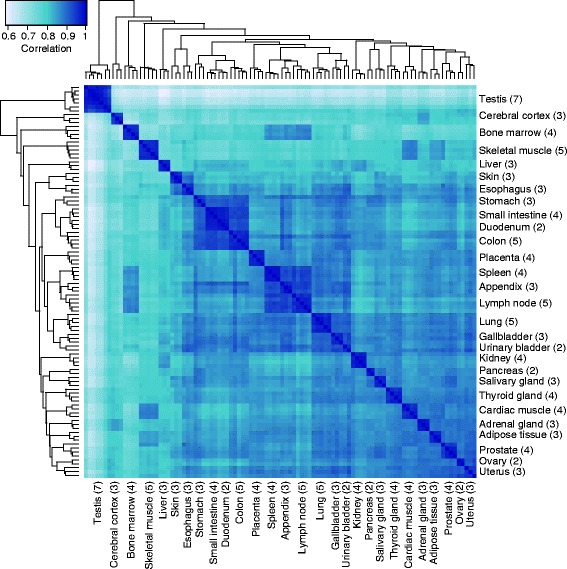
Hierarchical clustering of human tissues. The relationship between the 100 tissue samples corresponding to 28 different tissues, based on pairwise Spearman correlations

A transcriptomic analysis was performed using deep sequencing (RNA-seq) on five skeletal muscle samples. These were compared with our previously reported transcriptomic analysis on 27 fresh frozen tissue types including four heart muscle samples [4]. This resulted in altogether 100 samples corresponding to 28 tissue types. The normalized mRNA abundance was calculated as FPKM-values with a cutoff of FPKM = 1, roughly corresponding to one mRNA molecule per average cell in the sample [5]. The most abundant genes in muscle tissues were mitochondrial genes expressed in all tissue types, with the highest mRNA abundance (FPKM = 20,737) observed by the mitochondrial gene MT-CO1 in cardiac muscle. An analysis of the global expression profiles using hierarchical clustering and a correlation heat map from all the 100 tissue samples are displayed in Fig. 1. The heat map shows pairwise correlations between the 28 tissue types based on mRNA expression levels of all 20,314 genes. Testis and brain were identified as outliers, while a clear connectivity and similarities in global expression was observed between samples from the GI-tract, the hematopoietic tissues, as well as the two striated muscle tissues, respectively. Cardiac and skeletal muscle showed highest correlations with one another as compared to other tissue types.

**Fig. 2:**
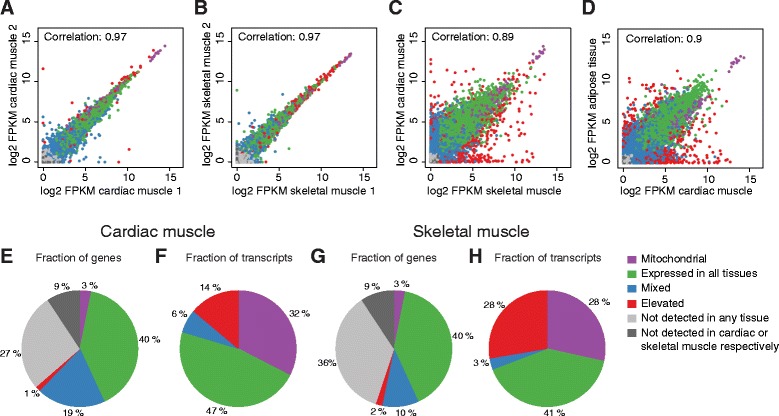
Sample correlations and classification of all human protein coding genes. Scatter plots of FPKM values for all detected genes in **a** two cardiac muscle samples, **b** two skeletal muscle samples, **c** cardiac muscle and skeletal muscle **d** and cardiac muscle and adipose tissue. **e** Pie chart showing the classification of all genes in cardiac muscle, based on transcript abundance and number of tissues with expression. **f** Pie chart showing the distribution of the expressed mRNA molecules in cardiac muscle. **g** Pie chart showing the classification of all genes in skeletal muscle. **h** Pie chart showing the distribution of expressed mRNA molecules in skeletal muscle

The variance between muscle samples from different individuals was analyzed by pairwise correlations of the expression level of all protein coding genes (Fig. 2a–d). A Spearman correlation of 0.97 was observed between cardiac muscle samples from two different individuals (Fig. 2a), while corresponding correlation for the skeletal muscle samples was also 0.97 (Fig. 2b). The correlation between average values from the two striated muscles was somewhat lower (Fig. 2c), but still at a high value (0.89). Low inter-individual variations in the genome-wide expression patterns across muscle samples indicate both high technical reproducibility and low biological variance. Pairwise comparisons between muscle tissues and other tissue types showed, as expected, higher variance. The lowest correlation was found between skeletal muscle and testis (0.69, data not shown), while a somewhat surprisingly high correlation (0.90) was observed between heart muscle and adipose tissue (Fig. 2d), as well as skeletal muscle and adipose tissue (0.86, data not shown). The similarity of global expression between adipose tissue and the striated muscle is interesting and should be explored further.

### The cardiac muscle transcriptome

Data from the transcriptomics analysis across 28 tissue types was used for categorization of all the protein-coding genes (*n* = 20,314) into various classes of tissue-specific expression. Figure 2e shows the classification for heart muscle. Altogether 64 % of the genes were expressed in the heart, with the largest class of genes (*n* = 8760) representing “housekeeping genes” expressed in all tissues, out of which 641 genes encoded mitochondrial proteins. Only 283 genes were defined as elevated in heart as compared to the other tissues (Additional file 1: Table S1), including genes enriched in a group of tissues. The 283 genes elevated in heart were classified into three subcategories as defined in Table 1, with 32 tissue enriched genes, 143 group enriched genes and 108 tissue enhanced genes. A GO-analysis of these genes indicated an over-representation of genes related to contraction (37 genes), ion transport (23 genes), regulation of membrane potential (22 genes) and actomyosin structure organization (18 genes).

**Table 1:** The genes with elevated expression in cardiac and/or skeletal muscle

Category	Cardiac muscle	Skeletal muscle	Description
Tissue enriched	32	110	Five-fold higher mRNA levels in specified muscle tissue as compared to other tissues
Group enriched	143	149	Five-fold higher mRNA levels in a group of 2–7 tissues, included specified muscle tissue
Tissue enhanced	108	112	Five-fold higher mRNA levels in specified muscle tissue as compared to average levels in all tissues
Elevated (total)	283	371	Total number of genes tissue enriched, group enriched or tissue enhanced in specified muscle tissue

Of the 283 genes elevated in cardiac muscle, 47 genes were differentially expressed between the two auricular samples and the two ventricle samples (Additional file 2: Table S2), based on mean values and a threshold of at least a five-fold difference in FPKM values. Twenty-one genes showed a higher average expression in the ventricle samples as compared to the auricle samples, while 26 genes were higher expressed in the auricle samples as compared to the ventricle samples. A majority of the differentially expressed genes encode proteins associated with developmental processes or regulatory functions such as control of blood volume, blood pressure, heart rate and response to cardiac overload. The list also includes several genes with unknown or only partly characterized functions in cardiac muscle.

An analysis of the expression levels of each gene made it possible to calculate the relative mRNA pool in heart for each category. The analysis (Fig. 2f) showed that 32 % of the mRNA molecules present in heart correspond to mitochondrial proteins and only 14 % of the mRNA pool represents genes elevated in heart. Thus, a large fraction of the transcriptional activity in the heart relates to generation of energy with almost one third of the transcripts encoding proteins implicated in energy metabolism.

### The skeletal muscle transcriptome

Figure 2g shows the classification for skeletal muscle. Altogether 55 % of the genes were expressed in skeletal muscle, with 8760 “housekeeping genes”. Only 371 genes (Additional file 3: Table S3) were defined as elevated in skeletal muscle as compared to other tissues, including 110 tissue enriched genes, 149 group enriched genes and 112 tissue enhanced genes. A GO-analysis of these genes indicated an over-representation of genes involved in contraction (48 genes), actin filament-based processes (37 genes), muscle organ development (24 genes) and muscle filament sliding (23 genes).

Analysis of the relative mRNA pool in skeletal muscle (Fig. 2h) showed that 28 % correspond to mitochondrial proteins and 28 % represent genes elevated in skeletal muscle. Thus, most of the transcriptional activity in the skeletal muscle also relates to energy generation, but with a higher fraction of mRNA molecules encoding genes classified as tissue elevated as compared to heart. Together the two striated muscles have by far the highest fraction of transcripts encoding mitochondrial proteins of all analyzed tissues (data not shown).

### Genes with shared enriched expression in cardiac and skeletal muscle

**Fig. 3:**
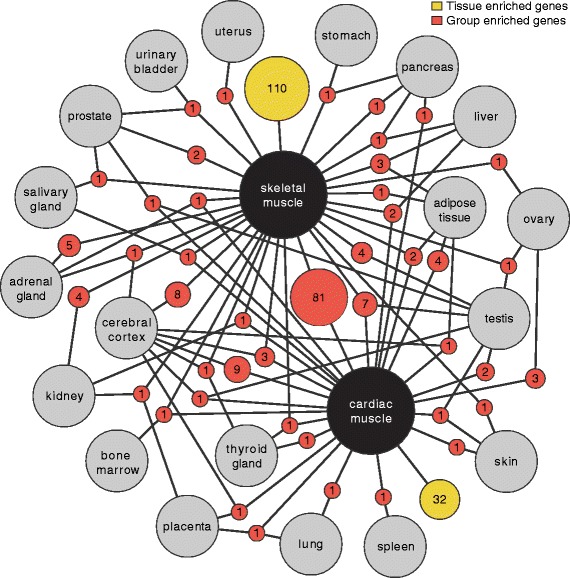
Network plot of the cardiac and skeletal muscle enriched and group enriched genes. Red circle nodes represent a group of expressed genes and are connected to the respective enriched tissues (grey circles). The size of each red node is related to the square root of the number of genes enriched in a particular combination of tissues

A network plot of the tissue and group enriched genes in heart and skeletal muscle, respectively, was generated in order to illustrate genes commonly expressed between different tissue types (Fig. 3). The two tissues had 81 simultaneously enriched genes, suggesting common functions. GO-analysis of these genes showed an overrepresentation of genes associated with contraction (18 genes), actin filament-based processes (15 genes), actomyosin structure organization (10 genes) and muscle filament sliding (8 genes). Several genes were also shared between striated muscles and cerebral cortex, and these genes are dominated by genes for which the corresponding proteins are involved in ion transport. Moreover, a number of genes were simultaneously enriched in striated muscles, adipose tissue and/or liver, mainly represented by genes implicated in metabolic processes and enzymatic activities.

### Proteins with elevated expression in cardiac muscle

The proteins elevated in heart or skeletal muscle identified by the transcriptomics analysis were further studied by antibody-based profiling using the publicly available resource of TMA data within the Human Protein Atlas (www.proteinatlas.org) portal cross-referenced with the Cardiac Organellar Protein Atlas Knowledge base (www.heartproteome.org) [6] in order to determine heart and skeletal muscle specificity and spatial and cellular distribution of the expression. In total, 239 of the 283 genes elevated in cardiac muscle, and 313 of the 371 genes elevated in skeletal muscle had available antibodies as part of the Human Protein Atlas project and were studied with antibody-based profiling using immunohistochemistry. Immunohistochemistry was performed on TMA cores from 44 different normal tissues, including heart and skeletal muscle samples from three different individuals, respectively. The analyzed proteins were divided into categories based on specificity of the expression, such as proteins selectively expressed in either heart or skeletal muscle, or proteins found to be expressed in both heart and skeletal muscle. Examples of proteins with various functions and expression patterns in striated muscles are described below.

**Fig. 4:**
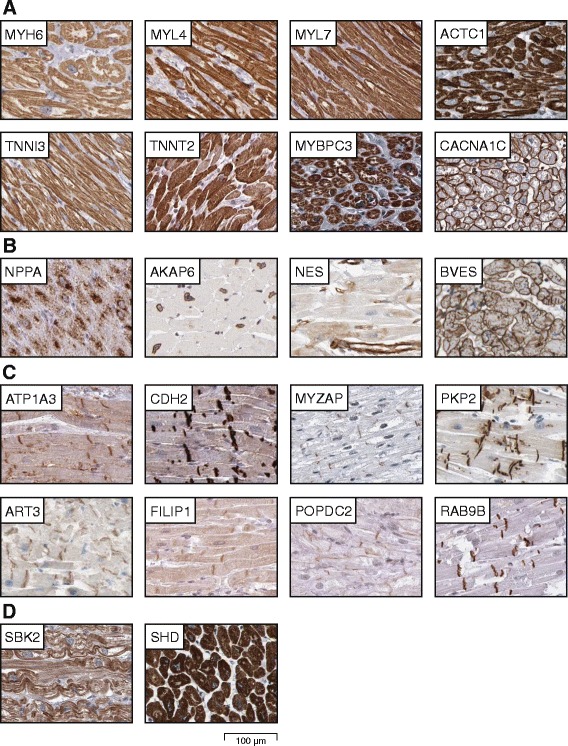
Immunohistochemical staining of proteins elevated in cardiac muscle. **a** Examples of eight proteins related to muscle contraction (MYH6, MYL4, MYL7, ACTC1, TNNI3, TNNT2, MYBPC3 and CACNA1C). CACNA1C was distinctly stained in sarcolemma and endothelial cells. **b** Examples of four proteins related to homeostasis and regeneration/repair, with NPPA displaying granular cytoplasmic expression, AKAP6 observed in nuclear membranes of cardiomyocytes, NES restricted to endothelial cells and BVES distinctly stained in sarcolemma. **c** Examples of eight proteins specific for intercalated discs (ATP1A3, CDH2, MYZAP, PKP2, ART3, FILIP1, POPDC2 and RAB9B). **d** Examples of two proteins with unknown function in cardiac muscle (SBK2 and SHD)

An interesting analysis is to investigate proteins elevated in heart, particularly in relation to skeletal muscle. Examples of eight proteins related to contraction and with highest expression in heart muscle are displayed in Fig. 4a. The primary structural proteins in the cardiomyocytes are myosin and actin filaments, related to contraction and forming a striated pattern observed in electron microscopy. Examples of members of the myosin and actin families mainly expressed in heart muscle include MYH6, MYL4, MYL7 and ACTC1. Another protein family related to muscular contraction is the troponin family, regulating the binding of myosin to actin via conformation differences dependent on calcium ion concentration in the cells [7]. Two troponin family members solely expressed in heart are TNNI3 and TNNT2. Other examples include the myosin binding protein MYBPC3, influencing contraction by cross-bridging in the sarcomere, and the calcium channel protein CACNA1C, regulating contraction via mediation of calcium ion entry into excitable cells.

A large number of proteins identified as highly expressed in heart were implicated in homeostasis and regeneration/repair, and some of these are displayed in Fig. 4b. In order to retain balanced levels of various substances in the body, heart plays an important role in homeostasis. One such example is the atrial natriuretic peptide NPPA, controlling extracellular fluid volume and electrolyte homeostasis. Mutations in NPPA are thought to be responsible for the development of atrial fibrillation (arrhythmia) [8]. Another example is AKAP6, involved in controlling energy homeostasis and glycolysis. Heart regeneration and repair have been extensively studied as cardiomyocyte deficiency is the underlying cause of most heart failures, and it is still debated as to what extent cardiomyocytes are replaced in adult hearts [9]. Examples of proteins expressed in heart suggested to play a role in regeneration and repair include the stem cell protein NES and the cell adhesion molecule BVES. NES is involved in brain and eye development and has been suggested to be involved in reparative vascularization and differentiation of cardiac stem cells into striated cells following myocardial infarction [10, 11]. NES displayed distinct positivity in endothelial cells of both heart and other tissue types. BVES is involved in formation and regulation of tight junction permeability and limited literature suggests involvement in muscle regeneration [12].

Intercalated discs are defined as the connections between two adjacent cardiomyocytes, aiding in contraction of multiple cardiomyocytes simultaneously as a unit, which is necessary for proper heart function. Examples of eight proteins distinctly expressed in intercalated discs are displayed in Fig. 4c. Four of these (ATP1A3, CDH2, MYZAP and PKP2) are well characterized in literature and involved in functions related to plasma membrane ion exchange, cell adhesion, cell junctions and desmosomes, respectively. For the remaining four proteins (ART3, FILIP1, POPDC2 and RAB9B) no or only limited literature exists on function in heart. ART3 has been suggested as an arginine-specific protein with function in testis [13], distinctly stained also in the present investigation, however, the additional high expression in intercalated discs has previously not been described. FILIP1, with evidence only at transcript level, is suggested to play a role in neuronal development and showed highest expression in heart and brain, but the exact function is unknown [14]. The transmembrane protein POPDC2 has in few studies on mice and zebrafish been suggested to play a role in proper sinus node function [15], but no previous studies suggest expression in intercalated discs. RAB9B has limited literature on membrane trafficking, but no data on expression in heart.

Interestingly, two proteins previously not characterized in heart (SBK2 and SHD) were at the mRNA level selectively expressed in both samples from the atrium and not in the ventricular wall or in any of the other analyzed tissues. Immunohistochemically stained images of SBK2 and SHD are displayed in Fig. 4d. SBK2 has evidence of existence only inferred from homology, while SHD is suggested to be an adapter protein with previous evidence only at transcript level.

### Proteins with elevated expression in skeletal muscle

**Fig. 5:**
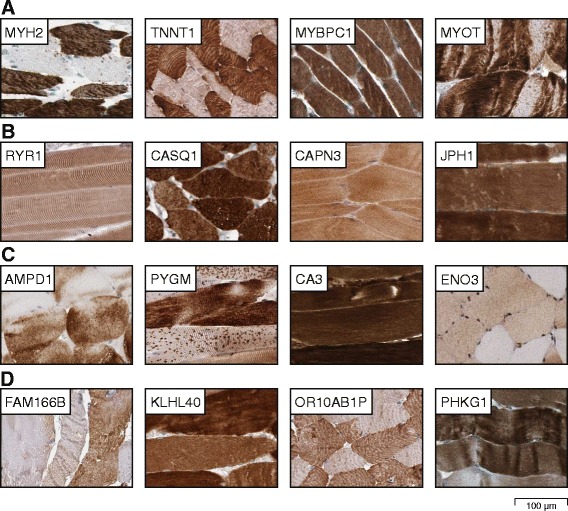
Immunohistochemical staining of proteins elevated in skeletal muscle. **a** Examples of four proteins related to muscle contraction (MYH2, TNNT1, MYBPC1 and MYOT). MYH2 and TNNT1 were differentially expressed between type I and type II muscle fibers, while MYOT concentrated to Z-lines of the muscle fibers. **b** Examples of four proteins related to calcium function (RYR1, CASQ1, CAPN3 and JPH1) (**c**) Examples of four proteins related to enzymatic activity (AMPD1, PYGM, CA3 and ENO3). AMPD1 and PYGM revealed heterogeneous cytoplasmic expression and ENO3 was differentially expressed between type I and type II muscle fibers. **d** Examples of four proteins with unknown function in skeletal muscle (FAM166B, KLHL40, OR10AB1P and PHKG1). Both OR10AB1P and PHKG1 being differentially expressed between type I and type II muscle fibers

Similar to heart muscle, the main function of skeletal muscle is contraction. Four proteins related to contraction with exclusive expression in skeletal muscle are displayed in Fig. 5a. Two of those (MYH2 and TNNT1) are members of the myosin and troponin families. Other examples include the myosin binding protein MYBPC1 and the actin cross-linking protein MYOT, involved in myofibril stability and assembly. MYH2 was expressed in fast (type II) fibers [16] and TNNT1 in slow (type I) fibers [17], while expression of MYOT was concentrated to Z-lines of the muscle fibers.

In both heart and skeletal muscle, contraction is dependent on the level of intracellular calcium. However, in contrast to cardiomyocytes, where calcium release is regulated via binding of external calcium ions to voltage gated calcium channels, skeletal myocytes store calcium in the sarcoplasmic reticulum until a neuronal impulse triggers calcium influx along the myofilaments. In Fig. 5b, four proteins related to calcium function with selective expression in skeletal muscle are displayed. Two of the main proteins responsible for this function are RYR1, the ryanodine receptor acting as the calcium release channel, and CASQ1, essential for calcium storage in the sarcoplasmic reticulum. CAPN3 is an intracellular protease regulated by changes in calcium levels and plays a role in skeletal muscle degradation and sarcomeric remodeling, with defects resulting in muscular dystrophy [18]. Another protein involved in calcium function is JPH1, aiding in the functional cross-talk between cell surface and intracellular calcium release channels.

Contraction is an energy demanding process and it is acknowledged that skeletal muscle is a tissue with high metabolic activity. Skeletal muscle is one of the body’s major sites for glucose disposal and is implicated in metabolic diseases such as type II diabetes. Thus, enzymatic activity is an important function in skeletal muscle, relating to various processes such as glucose metabolism, glycogen storage and regeneration. Indeed, a large number (72 out of 371) of the elevated proteins in skeletal muscle are related to different metabolic processes, i.e. present in the HMR2.0 metabolic reaction library [19, 20]. Genes from several enzymatic pathways were elevated in skeletal muscle, including membrane transport, protein modification and purine metabolism. The skeletal muscle specific transport reactions involved calcium transport (ATP2A1, ATP2B2, nucleoside uptake (SLC29A2) and glucose transport (SLCA4). Additional examples of proteins implicated in enzymatic activities with selective expression in skeletal muscle are displayed in Fig. 5c. These examples include (i) AMPD1, an enzyme involved in the purine nucleotide cycle, (ii) PYGM, an enzyme essential for carbohydrate metabolism and glycogenolysis, (iii) CA3, a metalloenzyme catalyzing hydration of carbon dioxide, and (iv) ENO3, an isoenzyme suggested to play a role in muscle development and regeneration, with mutations associated with glycogen storage disease [21]. At the protein level, ENO3 was highly expressed in type II fibers [22].

Four examples of proteins selectively expressed, but with unknown function in skeletal muscle are displayed in Fig. 5d. The putative protein FAM166B showed additional positivity in adrenal gland and ciliated cells in the present investigation. KLHL40 is suggested to be required for skeletal muscle development [23]; however, the exact function is unknown. Another example is OR10AB1P, with evidence of existence only at the transcript level according to Uniprot. Despite low FPKM values, OR10AB1P was selectively expressed in striated muscles as compared to other tissues, with strongest immunoreactivity observed in skeletal muscle. PHKG1 is the catalytic subunit of the phosphorylase b kinase (PHK), which is responsible for hormonal regulation of glycogen breakdown [24]; however, PHKG1 only has evidence of existence at the transcript level according to Uniprot and the exact function is unknown. FAM166B, OR10AB1P and PHKG1 displayed a heterogeneous expression pattern possibly representing type I or type II muscle fibers.

### Proteins with elevated expression in both cardiac and skeletal muscle

**Fig. 6:**
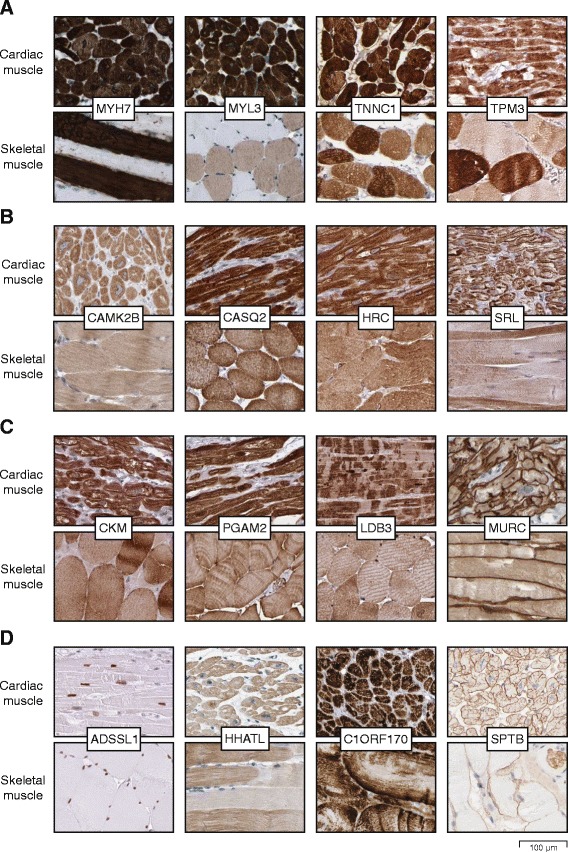
Immunohistochemical staining of proteins elevated in both cardiac and skeletal muscle. **a** Examples of four proteins related to muscular contraction (MYH7, MYL3, TNNC1 and TPM3), in skeletal muscle higher expressed in type I muscle fibers. **b** Examples of four proteins related to calcium transport and storage (CAMK2B, CASQ1, HRC and SRL). **c** Examples of four proteins related to metabolism and myofibrillar organization (CKM, PGAM2, LDB3 and MURC). LDB3 was restricted to Z-lines of cardiac muscle fibers while more homogeneous in skeletal muscle. MURC showed distinct expression of sarcolemma in both cardiac and skeletal muscle. **d** Examples of four proteins with unknown function in cardiac and skeletal muscle, with ADSSL1 showing distinct nuclear staining, HHATL and C1ORF170 displaying cytoplasmic positivity, and SPTB revealing distinct immunoreactivity in sarcolemma, accompanied with positivity in erythrocytes

Although heart and skeletal muscles differ in arrangement of cells and response to signals, the two muscle types have many similarities. Several proteins related to contraction are shared between the two organs, as displayed in Fig. 6a. Some of these are members of the myosin and troponin families, such as MYH7, MYL3 and TNNC1. Another example is TPM3, a member of the tropomyosin family essential for contraction. TPM3 binds to actin and is associated with the troponin complex. In skeletal muscle, all four proteins were higher expressed in type I muscle fibers.

In Fig. 6b, four proteins involved in calcium transport and storage expressed in both heart and skeletal muscle are shown. CAMK2B is involved in synapse formation and regulation of sarcoplasmic reticulum calcium transport [25]. Additional strong staining of CAMK2B was observed in the CNS. Other examples include CASQ2, responsible for the internal calcium store in muscle [26], HRC, implicated in regulation of calcium sequestration and release [27], and the calcium transport regulating protein SRL, with evidence of existence only at the transcript level according to Uniprot.

Examples of proteins involved in metabolism and myofibrillar organization with expression shared between heart and skeletal muscle are displayed in Fig. 6c. CKM is a creatine kinase involved in energy transduction, and is also an important serum marker for myocardial infarction [28]. Another enzyme is PGAM2, related to glycolytic pathways, with mutations causing glycogen storage disease [29]. LDB3 is involved in sarcomere organization, with mutations associated with myopathy [30]. MURC aids in myofibrillar organization and has also been shown to induce NPPA transcription [28, 31].

Four proteins with selective expression but unknown function in heart and skeletal muscle are displayed in Fig. 6d. The nuclear protein ADSSL1 has limited data suggesting contribution to glycolysis, while vague literature on HHATL mentions heart specificity [32], however, the exact function is unknown and neither ADSSL1 nor HHATL has previously been characterized on the protein level. Another protein only characterized on the transcript level is C1ORF170, which regulates the expression of target genes associated with energy transfer and contractile function [33]. SPTB is known to be involved in cytoskeletal structure of erythrocyte plasma membrane [34], and erythrocytes were distinctly stained also in the present investigation, however, the clear membranous expression in striated muscle cells has previously not been described.

## Discussion

To our knowledge, this is the first study utilizing an integrative omics approach comparing heart and skeletal muscle specific gene expression providing a detailed genome-wide transcriptomics overview characterizing normal striated muscles in relation to a broad range of other tissues. The tissue specific RNA levels were compared to corresponding *in situ* protein expression, by thorough visual examination of the immunohistochemical images provided by an antibody-based profiling approach. In this way, the muscle specific genes could be further stratified into different subcompartments present within the striated muscles. This allowed us to identify proteins with previously unknown patterns of expression, such as FILIP1 and POPDC2 distinctly expressed in intercalated discs of cardiac muscle. Using this approach, we were also able to identify novel proteins specifically expressed in myocytes and not present in other cell types in the samples, such as adipocytes, fibroblasts or endothelial cells, which suggests a role of these proteins in striated muscle physiology and function.

The immunohistochemistry-based profiling was performed within the infrastructure of the Human Protein Atlas program, a multidisciplinary research initiative which systematically generates and validates representative antibodies towards all non-redundant human proteins [35, 36]. In addition to performing immunohistochemistry on tissues with a well-established protocol, the antibodies are thoroughly tested in various other platforms such as protein arrays, Western blotting and cell lines, suggesting a high quality of the generated data. Data and high-resolution images from all approved antibodies together with the RNA-seq data for each gene are comprehensively published online at www.proteinatlas.org, which allows for further interpretation and analysis of the genes and proteins presented here.

The analysis of the heart muscle elevated proteins is well in line with the function of the heart. The list includes well-known proteins of the myosin, actin and troponin families, as well as the natriuretic peptides A and B, extensively studied as markers for heart failure [37]. In addition, several proteins previously not described in the context of heart were identified, such as the putative proteins SBK2 and SHD, selectively expressed in the atrium and not in the ventricle or in any of the other analyzed tissues. Other identified examples of proteins with yet unknown function include proteins expressed in intercalated discs, such as ART3, FILIP1, POPDC2 and RAB9B. The exact functions of these proteins remain to be elucidated, but given the selective expression in heart muscle at both the mRNA and protein level, an implication in heart physiology can be anticipated. Similarly, the analysis of skeletal muscle elevated proteins identified a large number of proteins with well-known function related to contraction, calcium storage and enzymatic activity, but also several proteins previously not characterized in skeletal muscle. As expected, a significant number of proteins were expressed in both heart and skeletal muscle, but our lists also included proteins unique for one of the organs. An interesting observation is the high correlation between striated muscles and adipose tissue, which may partly be explained by presence of adipocytes in the heart and skeletal muscle samples, but it possibly also has a functional relevance related to metabolic activities. Another interesting observation is that more than 30 % of the transcripts in heart and 28 % of the transcripts in skeletal muscle correspond to genes encoding mitochondrial proteins, which demonstrates the extreme specialization of both the heart and skeletal muscle to provide energy for the contraction. These two striated muscle tissues consequently have a higher fraction of mitochondrial transcripts than any of the other analyzed tissues. Mitochondrial genes were among the most abundant genes in striated muscles, and often showed FPKM values > 10,000. The natriuretic peptides A and B (NPPA and NPPB) and several genes associated with contraction were also relatively highly expressed. Interestingly, the lists of genes elevated in striated muscles included several genes with low mRNA abundance that were shown to be selectively expressed in cardiac or skeletal muscle also at the protein level, suggesting that these genes may have a regulatory function. Two such examples are BVES and RAB9B, distinctly expressed in membranes of cardiomyocytes and intercalated discs, respectively, both with FPKM values around 20.

It is important to point out that the heart samples used in the present investigation are taken from patients with various cardiovascular diseases and that the sample size is rather small. Although it cannot be ruled out that an underlying disease alters transcription of individual genes, the global expression profiles were shown to be highly similar between samples despite different disease background of the patients. Given that a large number of the genes identified as most abundant in heart represented genes associated with a well-known function and no overrepresentation of genes associated with other pathological conditions was observed, a good estimate of a normal mRNA profile can be anticipated.

From a medical point of view, the identification of the molecular constituents of the cardiac and skeletal muscle is important. Despite the high prevalence of myocardial infarction, the diagnosis remains a challenge as routine signs and symptoms have low specificity and sensitivity. Myocardial infarction may occur without symptoms, stressing the need for other diagnostic methods such as cardiac imaging or biomarker alterations. Research on serum-based biomarkers has identified several proteins that may serve as markers of cardiomyocyte injury, such as troponins, CKMB and MYL1 [3, 38]. Moreover, the natriuretic peptides NPPA and NPPB have been extensively studied as diagnostic and prognostic biomarkers in acute heart failure patients, showing promising results [37]. However, although alterations in these biomarkers may reflect myocardial necrosis or cardiac overload, more biomarkers are needed in order to identify and stratify high-risk individuals and further understand the underlying mechanisms. The data presented here can serve as a starting point for further biomarker discovery, since the gene expression repository can be used for identifying muscle specific biomarkers that are unique or common for different types of muscles.

## Conclusions

In summary, our work presents a comprehensive resource of proteins elevated in cardiac and skeletal muscle that have been identified and localized to specific muscle segments. This resource can be used for further molecular studies on striated muscle biology as well as for research studies comparing normal and pathological tissues.

## Methods

### Tissue samples

Tissues used for RNA extraction was based on biopsies from four heart muscle samples and five skeletal muscle samples. The heart material was composed of two samples from the left ventricle (both adult females) and two samples from the left auricles (one female and one male, both adults). One left ventricle sample was obtained by surgery for asymmetrical hypertrophy, with the sample showing slight fibrosis, but not diagnostic for hypertrophic cardiomyopathy. The other left ventricle sample was obtained from an individual diagnosed with post partum cardiomyopathy, with the material taken in connection with temporary use of left ventricle device (heart mate). The sample had some dilation and slight endocardial increased thickness. The histology of the sample was mainly normal, but also showed areas with increased fibrotic tissue and compensatory hypertrophy, as well as a slight probably unspecific inflammation. Both auricle samples were obtained from individuals operated for atrial fibrillation, with normal histological picture without amyloid depositions. The five skeletal muscle samples (two males and three females) were derived from the vastus lateralis muscle in adult healthy volunteers [39]. In addition, samples from 26 other tissues and organs were analyzed, as previously described [4].

### Transcript profiling (RNA-seq)

The use of human tissue samples was approved by the Uppsala Ethical Review Board (Reference #2011/473). Tissues samples were embedded in Optimal Cutting Temperature (O.C.T.) compound and stored at −80 °C. A hematoxylin-eosin (HE) stained frozen section (4 μm) was prepared from each sample using a cryostat and each slide was examined by a pathologist to ensure proper tissue morphology and sampling of representative normal tissue. Three sections (10 μm) were cut from each frozen tissue block and collected into a tube for subsequent RNA extraction. The tissue was homogenized mechanically using a 3 mm steel grinding ball (VWR). Total RNA was extracted from cell lines and tissue samples using the RNeasy Mini Kit (Qiagen, Hilden, Germany) according to the manufacturer’s instructions. For the skeletal muscle samples, RNA was extracted using the Trizol® method (Invitrogen, Carlsbad, CA, USA). The extracted RNA samples were analyzed using either an Experion automated electrophoresis system (Bio-Rad Laboratories, Hercules, CA, USA) with the standard-sensitivity RNA chip or an Agilent 2100 Bioanalyzer system (Agilent Biotechnologies, Palo Alto, USA) with the RNA 6000 Nano Labchip Kit. Only samples of high-quality RNA (RNA Integrity Number ≥7.5) were used in the following mRNA sample preparation for sequencing. mRNA sequencing was performed on Illumina HiSeq2000 and 2500 machines (Illumina, San Diego, CA, USA) using the standard Illumina RNA-seq protocol with a read length of 2 × 100 bases. The samples were sequenced multiplexed 15 per lane, producing an average of 18 million mappable read pairs per sample.

### Analysis of data

The raw reads obtained from the sequencing system were trimmed for low quality ends with the software Sickle (3), using a phred quality threshold of 20. All reads shorter than 54 bp after the trimming were discarded. The processed reads were mapped to the GRCh37 version of the human genome with Tophat v2.0.3 (4). Potential PCR duplicates were eliminated using the MarkDuplicates module of Picard 1.77 (5). To obtain quantification scores for all human genes and transcripts, FPKM (fragments per kilobase of exon model per million mapped reads) values were calculated using Cufflinks v2.0.2 (6), which corrects for transcript length and the total number of mapped reads from the library to compensate for different read depths for different samples. The gene models from Ensembl build 69 (7) were used in Cufflinks. Data was analyzed using R Statistical Environment (8) with the addition of package ‘gplots’ (9). A network analysis was performed using Cytoscape 3.0 (10). For analyses where a log2-scale of the data was used, pseudo-counts of +1 were added to the values.

### Specificity classification

The average FPKM value of all individual samples for each tissue was used to estimate the gene expression level and classify the 20,314 genes into one of six categories: (1) “Not detected”, <1 FPKM in tissue; (2) “Tissue enriched”, at least five-fold higher FPKM level in tissue compared to all other 27 tissues; (3) “Group enriched”, at least five-fold higher FPKM level in a group of 2–7 tissues compared to all other tissues; (4) “Expressed in all”, detected in all 28 tissues; (5) “Tissue enhanced”, at least five-fold higher FPKM level in tissue compared to the average FPKM value of all 28 tissues and (6) “Mixed”, genes expressed in 1–27 tissues and in none of the above categories. A tissue-specific score [4] was defined as the heart or skeletal muscle FPKM respectively, divided by the maximum FPKM value in any of the other 27 tissues. Genes categorized as tissue enriched, group enriched or tissue enhanced were together defined as tissue elevated, while genes in the “expressed in all” category were further classified into a “mitochondrial” category based on a list of mitochondrial proteins obtained from the MitoProteome database [40].

### Gene ontology analysis

A gene ontology (GO) [41] analysis was performed using the GOrilla tool [42] in order to determine overrepresented GO categories in the gene set of tissue elevated genes. The number of genes for each term was counted, allowing a gene to be associated with more than one term. A list of all genes analyzed in this study was used as the background list in GOrilla.

### Tissue profiling

Tissue microarrays (TMAs) were generated as previously described [43], containing 1 mm cores of 44 different normal tissues in triplicates. Formalin-fixed and paraffin-embedded samples were received from the Department of Pathology, Uppsala University Hospital, Sweden, approved by the local Research Ethics Committee (Uppsala, Sweden, Ups 02–577). One section (4 μm) was cut from each donor block, stained with HE and reviewed by a pathologist in order to select the appropriate area for sampling. Cylindrical cores with a diameter of 1 mm cylindrical tissue core were removed from the donor blocks and placed into recipient paraffin blocks, using an TMA Grand Master (3DHistech, Budapest, Hungary).

Four-micrometer sections were cut from the TMA blocks, mounted on adhesive slides and baked at 60 °C for 45 min. TMA slides were then deparaffinised in Neo-Clear® (Merck Millipore, Darmstadt, Germany), followed by hydration in graded alcohols and blocking for endogenous peroxidase in 0.3 % hydrogen peroxide. For antigen retrieval, slides were immersed and boiled in Citrate buffer®, pH6 (Lab Vision, Freemont, CA) for 4 min at 125 °C and then allowed to cool to 90 °C. Automated IHC was performed essentially as previously described [43], using an Autostainer 480 instrument® (Lab Vision). Primary antibodies and a dextran polymer visualization system (UltraVision LP HRP polymer®, Lab Vision) were incubated for 30 min each at room temperature and slides were developed for 10 min using Diaminobenzidine (Lab Vision) as chromogen. All incubations were followed by rinse in Wash buffer® (Lab Vision) for 5 min. Slides were counterstained in Mayers hematoxylin (Histolab) and cover slipped using Pertex® (Histolab) as mounting medium. Incubation with PBS instead of primary antibody served as negative control. The dilution of the primary antibody was optimized by testing the antibody on a selection of normal tissues and comparison with previously published literature and gene characterization data. The AperioScanScope XT Slide Scanner (Aperio Technologies, Vista, CA) system was used to capture digital whole slide high-resolution images with a 20× objective.

### Antibodies

The IDs of all antibodies used in the immunohistochemically stained images are summarized here. All antibodies are from the Human Protein Atlas project, unless otherwise stated. Proteins elevated in cardiac muscle: MYH6 (HPA001239), MYL4 (HPA051884), MYL7 (HPA013331), ACTC1 (61075, PROGEN Biotechnik), TNNI3 (HPA063258), TNNT2 (HPA015774), MYBPC3 (HPA040147), CACNA1C (HPA039796), NPPA (HPA058269), AKAP6 (07–087, Upstate), NES (HPA026111), BVES (HPA014788), ATP1A3 (HPA045367), CDH2 (sc-8424, Santa Cruz Biotechnology), MYZAP (HPA039827), PKP2 (HPA014314), ART3 (HPA011268), FILIP1 (HPA055754), POPDC2 (HPA024255), RAB9B (HPA061932), SBK2 (HPA030631), SHD (HPA017955). Proteins elevated in skeletal muscle: MYH2 (M4276, SIGMA), TNNT1 (HPA058448), MYBPC1 (HPA021004), MYOT (HPA037733), RYR1 (HPA056416), CASQ1 (HPA026823), CAPN3 (HPA040052), JPH1 (HPA009413), AMPD1 (HPA028080), PYGM (HPA056003), CA3 (H00000761-M02, Abnova), ENO3 (HPA000793), FAM166B (HPA045540), OR10AB1P (HPA044992), PHKG1 (HPA012057). Proteins elevated in both cardiac and skeletal muscle: MYH7 (sc-53090, Santa Cruz Biotechnology), MYL3 (sc-47719, Santa Cruz Biotechnology), TNNC1 (NCL-TROPC, Novocastra), TPM3 (HPA047089), CAMK2B (HPA026307), CASQ2 (HPA027285), HRC (HPA004833), SRL (HPA041535), CKM (HPA047859), PGAM2 (HPA060173), LDB3 (HPA048955), MURC (HPA020973), ADSSL1 (HPA052621), HHATL (HPA018174), C1ORF170 (HPA031712), SPTB (HPA003398).

### Data availability

FPKM values for samples from all analyzed tissues except skeletal muscle are available for download without any restrictions (www.proteinatlas.org/about/download). The skeletal muscle data will be added to the next release of the Human Protein Atlas. The primary data (reads) are available through the Array Express Archive (www.ebi.ac.uk/arrayexpress/) under accession number E-MTAB-1733, as well as at Gene Expression Omnibus under the accession numbers GSE58387 and GSE58608. Transcript profiling data for each gene in each tissue type, as well as the original immunohistochemically stained images are also available on the Human Protein Atlas (www.proteinatlas.org).

## Additional files

Additional file 1: Table S1. List of the 283 genes elevated in cardiac muscle. The list is sorted after tissue-specific score which is the FPKM value in cardiac muscle divided by the maximum FPKM in all other tissues. Additional file 2: Table S2. List of 47 genes elevated in cardiac muscle and differentially expressed between ventricle and auricle samples. The list includes genes showing at least a five-fold difference between mean FPKM values of ventricle and auricle samples, respectively. Additional file 3: Table S3. List of the 371 genes elevated in skeletal muscle. The list is sorted after tissue-specific score which is the FPKM value in skeletal muscle divided by the maximum FPKM in all other tissues.

## Abbreviations

FPKM: Fragments per kilobase of exon model per million mapped reads
GO: Gene ontology
TMA: Tissue microarray
