# Supplementary material for: The human cardiac and skeletal muscle proteomes defined by transcriptomics and antibody-based profiling
Source: BMC Genomics. 2015 Jun 25;16(1):475. doi: 10.1186/s12864-015-1686-y (PMC4479346; doi:10.1186/s12864-015-1686-y)
Supplement: Additional file 1: Table S1. — List of the 283 genes elevated in cardiac muscle. The list is sorted after tissue-specific score which is the FPKM value in cardiac muscle divided by the maximum FPKM in all other tissues. [file 12864_2015_1686_MOESM1_ESM.docx]

**Supplemental Table 1. List of the 283 genes elevated in cardiac muscle.** The list is sorted after tissue-specific score which is the FPKM value in cardiac muscle divided by the maximum FPKM in all other tissues.

| **Gene** | **Description** | **mRNA level (FPKM) Sample 1** | **mRNA level (FPKM) Sample 2** | **mRNA level (FPKM) Sample 3** | **mRNA level (FPKM) Sample 4** | **Mean**  **mRNA level (FPKM)** | **Tissue-specific score** | **Category** | **Number of antibodies with tissue data at www.proteinatlas.org** |
| --- | --- | --- | --- | --- | --- | --- | --- | --- | --- |
| NPPB | natriuretic peptide B | 3816 | 566 | 1470 | 3521 | 2343 | 2076.3 | Enriched | 0 |
| MYL7 | myosin, light chain 7, regulatory | 3108 | 603 | 6351 | 8365 | 4607 | 597.4 | Enriched | 3 |
| NPPA | natriuretic peptide A | 2056 | 1070 | 8928 | 14719 | 6693 | 353.5 | Enriched | 1 |
| TNNI3 | troponin I type 3 | 4122 | 1860 | 1086 | 1563 | 2157 | 335.0 | Enriched | 1 |
| LRRC10 | leucine rich repeat containing 10 | 41 | 21 | 17 | 30 | 27 | 246.4 | Enriched | 2 |
| MYBPC3 | myosin binding protein C, cardiac | 0 | 0 | 513 | 0 | 128 | 172.4 | Enriched | 2 |
| ANKRD1 | ankyrin repeat domain 1 | 3583 | 2336 | 1774 | 2113 | 2452 | 107.4 | Enriched | 1 |
| TNNT2 | troponin T type 2 | 3029 | 2880 | 2556 | 2310 | 2694 | 100.0 | Enriched | 3 |
| RD3L | retinal degeneration 3-like | 29 | 14 | 13 | 18 | 19 | 71.8 | Enriched | 0 |
| MYH6 | myosin, heavy chain 6, cardiac muscle, alpha | 60 | 53 | 0 | 3022 | 784 | 67.7 | Enriched | 3 |
| BMP10 | bone morphogenetic protein 10 | 0 | 0 | 1207 | 2 | 302 | 57.0 | Enriched | 0 |
| MYBPHL | myosin binding protein H-like | 0 | 0 | 165 | 189 | 89 | 56.4 | Enriched | 0 |
| SBK2 | SH3-binding domain kinase family, member 2 | 0 | 0 | 27 | 53 | 20 | 54.9 | Enriched | 1 |
| SCN5A | sodium channel, voltage-gated, type V, alpha subunit | 29 | 29 | 26 | 42 | 32 | 44.4 | Enriched | 0 |
| TECRL | trans-2,3-enoyl-CoA reductase-like | 930 | 1203 | 470 | 614 | 804 | 34.8 | Enriched | 0 |
| MYL4 | myosin, light chain 4, alkali; atrial, embryonic | 63 | 149 | 1914 | 1779 | 976 | 19.0 | Enriched | 1 |
| TNNI3K | TNNI3 interacting kinase | 62 | 91 | 41 | 38 | 58 | 14.6 | Enriched | 0 |
| CHRNE | cholinergic receptor, nicotinic, epsilon | 0 | 0 | 36 | 112 | 37 | 14.4 | Enriched | 0 |
| RYR2 | ryanodine receptor 2 | 57 | 55 | 113 | 106 | 83 | 14.2 | Enriched | 3 |
| CCDC141 | coiled-coil domain containing 141 | 35 | 76 | 50 | 98 | 65 | 13.7 | Enriched | 1 |
| DAND5 | DAN domain family member 5, BMP antagonist | 8 | 7 | 7 | 5 | 7 | 13.2 | Enriched | 1 |
| ACTC1 | actin, alpha, cardiac muscle 1 | 2687 | 2510 | 3077 | 3383 | 2914 | 11.6 | Enriched | 4 |
| POPDC2 | popeye domain containing 2 | 366 | 425 | 266 | 372 | 357 | 11.5 | Enriched | 1 |
| CASQ2 | calsequestrin 2 | 741 | 887 | 468 | 543 | 660 | 9.2 | Enriched | 3 |
| MYZAP | myocardial zonula adherens protein | 447 | 391 | 382 | 441 | 415 | 8.8 | Enriched | 1 |
| CORIN | corin, serine peptidase | 1 | 31 | 98 | 125 | 63 | 7.7 | Enriched | 0 |
| MYOZ2 | myozenin 2 | 563 | 592 | 319 | 405 | 470 | 7.2 | Enriched | 2 |
| SGK110 | putative uncharacterized serine/threonine-protein kinase SgK110 | 0 | 1 | 32 | 28 | 15 | 6.3 | Enriched | 0 |
| TENM2 | teneurin transmembrane protein 2 | 34 | 59 | 35 | 10 | 35 | 5.6 | Enriched | 2 |
| TBX20 | T-box 20 | 11 | 23 | 49 | 23 | 26 | 5.6 | Enriched | 1 |
| SLC8A1 | solute carrier family 8 member 1 | 102 | 129 | 104 | 120 | 114 | 5.0 | Enriched | 1 |
| RBPMS2 | RNA binding protein with multiple splicing 2 | 101 | 95 | 170 | 187 | 138 | 5.0 | Enriched | 1 |
| MTUS2 | microtubule associated tumor suppressor candidate 2 | 36 | 53 | 29 | 46 | 41 | 4.8 | Enhanced | 2 |
| LRRC20 | leucine rich repeat containing 20 | 29 | 46 | 20 | 27 | 31 | 4.8 | Group enr | 1 |
| TTN | Titin | 79 | 48 | 43 | 54 | 56 | 4.8 | Group enr | 2 |
| FGF12 | fibroblast growth factor 12 | 19 | 381 | 334 | 445 | 295 | 4.8 | Enhanced | 0 |
| PXDNL | peroxidasin homolog (Drosophila)-like | 31 | 40 | 28 | 25 | 31 | 4.7 | Group enr | 1 |
| PLN | Phospholamban | 1409 | 1136 | 903 | 1459 | 1227 | 4.7 | Group enr | 2 |
| FABP3 | fatty acid binding protein 3, muscle and heart | 3151 | 1750 | 1644 | 1848 | 2098 | 4.6 | Group enr | 2 |
| FGF18 | fibroblast growth factor 18 | 15 | 12 | 1 | 1 | 7 | 4.6 | Enhanced | 1 |
| RPL3L | ribosomal protein L3-like | 106 | 78 | 17 | 57 | 64 | 4.6 | Group enr | 1 |
| ADCY5 | adenylate cyclase 5 | 53 | 70 | 94 | 111 | 82 | 4.5 | Enhanced | 1 |
| ASB2 | ankyrin repeat and SOCS box containing 2 | 48 | 45 | 27 | 42 | 40 | 4.5 | Group enr | 1 |
| CMYA5 | cardiomyopathy associated 5 | 178 | 139 | 161 | 145 | 156 | 4.4 | Group enr | 0 |
| ABRA | actin-binding Rho activating protein | 12 | 14 | 13 | 13 | 13 | 4.3 | Group enr | 1 |
| CAV3 | caveolin 3 | 25 | 15 | 12 | 16 | 17 | 4.2 | Group enr | 2 |
| MLIP | muscular LMNA-interacting protein | 193 | 299 | 111 | 163 | 191 | 3.9 | Group enr | 1 |
| NT5C1A | 5'-nucleotidase, cytosolic IA | 4 | 9 | 10 | 6 | 7 | 3.8 | Group enr | 2 |
| FITM2 | fat storage-inducing transmembrane protein 2 | 24 | 51 | 46 | 67 | 47 | 3.8 | Enhanced | 0 |
| MYLK3 | myosin light chain kinase 3 | 26 | 41 | 105 | 136 | 77 | 3.7 | Group enr | 1 |
| FILIP1 | filamin A interacting protein 1 | 42 | 45 | 38 | 48 | 43 | 3.7 | Enhanced | 1 |
| LRRC14B | leucine rich repeat containing 14B | 10 | 2 | 8 | 13 | 8 | 3.7 | Group enr | 2 |
| KCNA5 | potassium voltage-gated channel, shaker-related subfamily, member 5 | 6 | 1 | 33 | 32 | 18 | 3.6 | Enhanced | 2 |
| OXCT1 | 3-oxoacid CoA transferase 1 | 289 | 293 | 116 | 208 | 226 | 3.6 | Enhanced | 1 |
| FHOD3 | formin homology 2 domain containing 3 | 152 | 165 | 56 | 70 | 111 | 3.6 | Enhanced | 2 |
| ZFP57 | ZFP57 zinc finger protein | 11 | 6 | 9 | 0 | 6 | 3.5 | Enhanced | 1 |
| PDE1C | phosphodiesterase 1C, calmodulin-dependent 70kDa | 50 | 61 | 45 | 41 | 49 | 3.5 | Enhanced | 2 |
| PTPLA | protein tyrosine phosphatase-like member A | 75 | 78 | 37 | 43 | 58 | 3.4 | Enhanced | 0 |
| C10orf71 | chromosome 10 open reading frame 71 | 41 | 34 | 32 | 33 | 35 | 3.3 | Group enr | 1 |
| DKK3 | dickkopf WNT signaling pathway inhibitor 3 | 289 | 447 | 1365 | 1724 | 956 | 3.3 | Enhanced | 2 |
| TRIM54 | tripartite motif containing 54 | 107 | 94 | 54 | 60 | 79 | 3.3 | Group enr | 1 |
| HSPB3 | heat shock 27kDa protein 3 | 188 | 203 | 307 | 289 | 247 | 3.3 | Group enr | 0 |
| PKP2 | plakophilin 2 | 183 | 231 | 112 | 164 | 173 | 3.1 | Enhanced | 1 |
| ITGB1BP2 | integrin beta 1 binding protein 2 | 61 | 62 | 81 | 87 | 73 | 3.1 | Group enr | 0 |
| FPGT-TNNI3K | FPGT-TNNI3K readthrough | 6 | 7 | 8 | 3 | 6 | 3.0 | Enhanced | 0 |
| MYO18A | myosin XVIIIA | 179 | 106 | 126 | 105 | 129 | 3.0 | Group enr | 2 |
| XIRP2 | xin actin-binding repeat containing 2 | 271 | 79 | 8 | 19 | 94 | 3.0 | Group enr | 1 |
| NRAP | nebulin-related anchoring protein | 326 | 228 | 143 | 194 | 223 | 3.0 | Group enr | 2 |
| APOBEC2 | apolipoprotein B mRNA editing enzyme, catalytic polypeptide-like 2 | 116 | 179 | 99 | 125 | 129 | 3.0 | Group enr | 2 |
| ANKRD2 | ankyrin repeat domain 2 | 61 | 68 | 5 | 10 | 36 | 2.9 | Group enr | 2 |
| ST8SIA2 | ST8 alpha-N-acetyl-neuraminide alpha-2,8-sialyltransferase 2 | 7 | 5 | 0 | 1 | 3 | 2.9 | Enhanced | 1 |
| NKX2-5 | NK2 homeobox 5 | 20 | 23 | 36 | 40 | 30 | 2.9 | Group enr | 0 |
| NES | Nestin | 120 | 72 | 116 | 147 | 114 | 2.9 | Enhanced | 4 |
| MURC | muscle-related coiled-coil protein | 48 | 63 | 16 | 22 | 37 | 2.9 | Group enr | 3 |
| RCAN2 | regulator of calcineurin 2 | 214 | 368 | 131 | 190 | 226 | 2.8 | Enhanced | 1 |
| CSRP3 | cysteine and glycine-rich protein 3 | 1462 | 1307 | 867 | 1114 | 1187 | 2.8 | Group enr | 1 |
| SH3BGR | SH3 domain binding glutamic acid-rich protein | 200 | 130 | 40 | 51 | 105 | 2.8 | Group enr | 2 |
| COLQ | collagen-like tail subunit of asymmetric acetylcholinesterase | 11 | 6 | 16 | 30 | 16 | 2.7 | Enhanced | 0 |
| HHATL | hedgehog acyltransferase-like | 478 | 331 | 349 | 480 | 409 | 2.7 | Group enr | 1 |
| EEF1A2 | eukaryotic translation elongation factor 1 alpha 2 | 456 | 383 | 242 | 328 | 352 | 2.7 | Enhanced | 4 |
| CAND2 | cullin-associated and neddylation-dissociated 2 | 19 | 18 | 15 | 22 | 19 | 2.6 | Group enr | 1 |
| PFKM | phosphofructokinase, muscle | 275 | 264 | 154 | 179 | 218 | 2.6 | Group enr | 1 |
| RP11-849F2.7 | KRAB-A domain-containing protein 2 | 1 | 1 | 3 | 2 | 2 | 2.6 | Enhanced | 0 |
| FLNC | filamin C, gamma | 198 | 193 | 59 | 75 | 131 | 2.6 | Enhanced | 1 |
| LSMEM2 | leucine-rich single-pass membrane protein 2 | 21 | 31 | 8 | 11 | 18 | 2.6 | Group enr | 1 |
| SLC4A3 | solute carrier family 4, anion exchanger, member 3 | 83 | 129 | 131 | 217 | 140 | 2.6 | Group enr | 1 |
| SRPK3 | SRSF protein kinase 3 | 27 | 37 | 24 | 18 | 26 | 2.5 | Group enr | 1 |
| PDE3A | phosphodiesterase 3A, cGMP-inhibited | 30 | 57 | 59 | 61 | 52 | 2.5 | Enhanced | 1 |
| CHRM2 | cholinergic receptor, muscarinic 2 | 19 | 34 | 34 | 54 | 35 | 2.4 | Enhanced | 2 |
| FITM1 | fat storage-inducing transmembrane protein 1 | 43 | 39 | 34 | 83 | 50 | 2.4 | Group enr | 1 |
| LRRC49 | leucine rich repeat containing 49 | 99 | 63 | 20 | 14 | 49 | 2.4 | Enhanced | 1 |
| TRIM55 | tripartite motif containing 55 | 54 | 43 | 48 | 51 | 49 | 2.4 | Group enr | 2 |
| KLHL38 | kelch-like family member 38 | 34 | 18 | 45 | 47 | 36 | 2.4 | Group enr | 2 |
| POPDC3 | popeye domain containing 3 | 46 | 47 | 22 | 39 | 38 | 2.4 | Group enr | 1 |
| CDH19 | cadherin 19, type 2 | 6 | 12 | 15 | 14 | 12 | 2.3 | Enhanced | 1 |
| ASB15 | ankyrin repeat and SOCS box containing 15 | 14 | 23 | 23 | 24 | 21 | 2.3 | Group enr | 1 |
| NEBL | Nebulette | 238 | 274 | 245 | 302 | 265 | 2.3 | Enhanced | 2 |
| CDH2 | cadherin 2, type 1, N-cadherin | 152 | 190 | 133 | 113 | 147 | 2.3 | Enhanced | 3 |
| PGAM2 | phosphoglycerate mutase 2 | 456 | 276 | 542 | 892 | 542 | 2.3 | Group enr | 2 |
| ADPRHL1 | ADP-ribosylhydrolase like 1 | 190 | 319 | 220 | 294 | 256 | 2.3 | Group enr | 1 |
| RBM20 | RNA binding motif protein 20 | 40 | 32 | 30 | 26 | 32 | 2.2 | Enhanced | 2 |
| MYPN | myopalladin | 60 | 52 | 43 | 38 | 48 | 2.2 | Group enr | 1 |
| HFE2 | hemochromatosis type 2 | 38 | 58 | 57 | 58 | 53 | 2.2 | Group enr | 1 |
| HRC | histidine rich calcium binding protein | 423 | 430 | 298 | 275 | 357 | 2.2 | Group enr | 1 |
| AC117834.1 | N/A | 4 | 3 | 0 | 1 | 2 | 2.2 | Enhanced | 0 |
| KBTBD12 | kelch repeat and BTB domain containing 12 | 18 | 20 | 3 | 2 | 11 | 2.2 | Group enr | 1 |
| METTL11B | methyltransferase like 11B | 1 | 2 | 1 | 2 | 1 | 2.2 | Enhanced | 1 |
| PPAPDC3 | phosphatidic acid phosphatase type 2 domain containing 3 | 40 | 27 | 29 | 47 | 36 | 2.2 | Group enr | 1 |
| COL21A1 | collagen, type XXI, alpha 1 | 48 | 57 | 31 | 33 | 42 | 2.2 | Enhanced | 3 |
| CRYAB | crystallin, alpha B | 3659 | 2585 | 2255 | 2144 | 2661 | 2.1 | Enhanced | 3 |
| CRYM | crystallin, mu | 208 | 79 | 111 | 100 | 125 | 2.1 | Enhanced | 1 |
| COX6A2 | cytochrome c oxidase subunit VIa polypeptide 2 | 1193 | 516 | 300 | 859 | 717 | 2.1 | Group enr | 0 |
| HSPB7 | heat shock 27kDa protein family, member 7 | 1286 | 1918 | 652 | 1197 | 1263 | 2.1 | Group enr | 1 |
| DHRS7C | dehydrogenase/reductase member 7C | 1 | 13 | 114 | 135 | 66 | 2.1 | Group enr | 2 |
| GJA3 | gap junction protein, alpha 3, 46kDa | 8 | 8 | 3 | 5 | 6 | 2.1 | Enhanced | 0 |
| PRKAA2 | protein kinase, AMP-activated, alpha 2 catalytic subunit | 33 | 29 | 29 | 38 | 32 | 2.1 | Enhanced | 1 |
| PTP4A3 | protein tyrosine phosphatase type IVA, member 3 | 174 | 128 | 85 | 135 | 130 | 2.1 | Group enr | 1 |
| ALPK2 | alpha-kinase 2 | 21 | 42 | 36 | 43 | 35 | 2.0 | Enhanced | 4 |
| SMCO1 | single-pass membrane protein with coiled-coil domains 1 | 45 | 30 | 21 | 32 | 32 | 2.0 | Group enr | 1 |
| LMOD3 | leiomodin 3 | 85 | 92 | 54 | 103 | 83 | 2.0 | Group enr | 1 |
| FRMD3 | FERM domain containing 3 | 88 | 101 | 40 | 46 | 69 | 2.0 | Enhanced | 1 |
| XIRP1 | xin actin-binding repeat containing 1 | 213 | 139 | 73 | 86 | 128 | 2.0 | Group enr | 3 |
| TRDN | Triadin | 273 | 284 | 142 | 172 | 218 | 2.0 | Group enr | 1 |
| SPHKAP | SPHK1 interactor, AKAP domain containing | 20 | 23 | 27 | 23 | 23 | 2.0 | Group enr | 2 |
| RAB9B | RAB9B, member RAS oncogene family | 16 | 25 | 11 | 17 | 17 | 1.9 | Enhanced | 1 |
| LRRC39 | leucine rich repeat containing 39 | 112 | 82 | 73 | 105 | 93 | 1.9 | Group enr | 2 |
| TSPAN9 | tetraspanin 9 | 113 | 171 | 138 | 109 | 133 | 1.9 | Enhanced | 1 |
| SORBS2 | sorbin and SH3 domain containing 2 | 737 | 1345 | 457 | 521 | 765 | 1.9 | Enhanced | 2 |
| PPP1R3A | protein phosphatase 1, regulatory subunit 3A | 30 | 39 | 27 | 35 | 33 | 1.9 | Group enr | 0 |
| SGCA | sarcoglycan, alpha | 112 | 104 | 67 | 109 | 98 | 1.9 | Group enr | 1 |
| PLA2G5 | phospholipase A2, group V | 41 | 64 | 78 | 73 | 64 | 1.9 | Enhanced | 1 |
| TBX5 | T-box 5 | 14 | 20 | 68 | 83 | 46 | 1.9 | Group enr | 1 |
| MYOM2 | myomesin 2 | 586 | 332 | 397 | 349 | 416 | 1.8 | Group enr | 2 |
| NMRK2 | nicotinamide riboside kinase 2 | 382 | 253 | 291 | 464 | 347 | 1.8 | Group enr | 1 |
| PTGES3L | prostaglandin E synthase 3 (cytosolic)-like | 43 | 44 | 25 | 38 | 38 | 1.8 | Group enr | 0 |
| TXLNB | taxilin beta | 67 | 72 | 47 | 57 | 61 | 1.8 | Group enr | 3 |
| DCAF8 | DDB1- and CUL4-associated factor 8 | 0 | 0 | 4 | 6 | 3 | 1.8 | Enhanced | 3 |
| RP11-650K20.3 | Uncharacterized protein | 1 | 3 | 1 | 1 | 1 | 1.8 | Enhanced | 0 |
| CTNNA3 | catenin, alpha 3 | 28 | 34 | 10 | 18 | 22 | 1.8 | Enhanced | 1 |
| GSG1L | GSG1-like | 0 | 2 | 21 | 19 | 10 | 1.7 | Enhanced | 1 |
| S100A1 | S100 calcium binding protein A1 | 2105 | 1521 | 1191 | 1310 | 1532 | 1.7 | Group enr | 2 |
| AKAP6 | A kinase anchor protein 6 | 39 | 62 | 53 | 67 | 55 | 1.7 | Enhanced | 2 |
| COX7A1 | cytochrome c oxidase subunit VIIa polypeptide 1 | 543 | 374 | 480 | 501 | 474 | 1.7 | Group enr | 0 |
| LRRC2 | leucine rich repeat containing 2 | 39 | 53 | 21 | 32 | 37 | 1.7 | Group enr | 2 |
| GPR22 | G protein-coupled receptor 22 | 20 | 20 | 21 | 18 | 20 | 1.7 | Enhanced | 1 |
| SYNPO2L | synaptopodin 2-like | 236 | 159 | 118 | 208 | 180 | 1.7 | Group enr | 2 |
| TCAP | titin-cap | 2165 | 1969 | 820 | 1484 | 1609 | 1.7 | Group enr | 2 |
| KLHL31 | kelch-like family member 31 | 28 | 44 | 29 | 35 | 34 | 1.7 | Enhanced | 1 |
| KCNE1 | potassium voltage-gated channel, Isk-related family, member 1 | 10 | 9 | 7 | 7 | 8 | 1.7 | Enhanced | 1 |
| CACNA1C | calcium channel, voltage-dependent, L type, alpha 1C subunit | 25 | 33 | 38 | 27 | 31 | 1.7 | Enhanced | 1 |
| COQ10A | coenzyme Q10 homolog A | 126 | 93 | 77 | 83 | 95 | 1.6 | Group enr | 0 |
| CRIP3 | cysteine-rich protein 3 | 47 | 26 | 4 | 5 | 20 | 1.6 | Enhanced | 1 |
| VWC2 | von Willebrand factor C domain containing 2 | 29 | 20 | 14 | 18 | 20 | 1.6 | Group enr | 1 |
| HAND1 | heart and neural crest derivatives expressed 1 | 14 | 8 | 1 | 3 | 7 | 1.6 | Group enr | 1 |
| LDB3 | LIM domain binding 3 | 557 | 849 | 452 | 470 | 582 | 1.6 | Group enr | 1 |
| SGCG | sarcoglycan, gamma | 130 | 95 | 74 | 117 | 104 | 1.6 | Enhanced | 2 |
| FSD2 | fibronectin type III and SPRY domain containing 2 | 20 | 32 | 44 | 40 | 34 | 1.6 | Group enr | 1 |
| TMEM71 | transmembrane protein 71 | 38 | 64 | 17 | 26 | 36 | 1.6 | Enhanced | 1 |
| QRFPR | pyroglutamylated RFamide peptide receptor | 0 | 2 | 1 | 0 | 1 | 1.6 | Enhanced | 1 |
| DOK7 | docking protein 7 | 23 | 12 | 7 | 14 | 14 | 1.5 | Enhanced | 1 |
| LMOD2 | leiomodin 2 | 641 | 746 | 278 | 309 | 493 | 1.5 | Group enr | 1 |
| RBM24 | RNA binding motif protein 24 | 126 | 82 | 107 | 148 | 116 | 1.5 | Enhanced | 0 |
| SMYD1 | SET and MYND domain containing 1 | 109 | 206 | 59 | 82 | 114 | 1.5 | Group enr | 1 |
| ATP1A2 | ATPase, Na+/K+ transporting, alpha 2 polypeptide | 81 | 61 | 75 | 63 | 70 | 1.5 | Group enr | 1 |
| CKMT2 | creatine kinase, mitochondrial 2 | 922 | 903 | 571 | 647 | 760 | 1.5 | Group enr | 1 |
| SPTB | spectrin, beta, erythrocytic | 17 | 16 | 18 | 20 | 18 | 1.5 | Group enr | 4 |
| ASB11 | ankyrin repeat and SOCS box containing 11 | 30 | 52 | 17 | 49 | 37 | 1.5 | Group enr | 1 |
| CXorf31 | chromosome X open reading frame 31 | 0 | 3 | 0 | 0 | 1 | 1.5 | Enhanced | 0 |
| TUBA8 | tubulin, alpha 8 | 85 | 32 | 96 | 146 | 90 | 1.5 | Group enr | 3 |
| SGCD | sarcoglycan, delta | 29 | 72 | 37 | 46 | 46 | 1.4 | Enhanced | 1 |
| TPM1 | tropomyosin 1 | 7541 | 5351 | 3063 | 3795 | 4938 | 1.4 | Group enr | 3 |
| ALPK3 | alpha-kinase 3 | 47 | 45 | 51 | 51 | 48 | 1.4 | Group enr | 1 |
| TMEM182 | transmembrane protein 182 | 49 | 105 | 82 | 112 | 87 | 1.4 | Group enr | 1 |
| SLC2A4 | solute carrier family 2, member 4 | 54 | 67 | 58 | 75 | 63 | 1.4 | Enhanced | 1 |
| ANO5 | anoctamin 5 | 15 | 18 | 19 | 26 | 20 | 1.4 | Enhanced | 1 |
| PALM2 | paralemmin 2 | 19 | 23 | 5 | 6 | 13 | 1.4 | Enhanced | 0 |
| PLCXD3 | phosphatidylinositol-specific phospholipase C, X domain containing 3 | 27 | 35 | 19 | 28 | 27 | 1.4 | Enhanced | 1 |
| DCUN1D2 | DCN1, defective in cullin neddylation 1, domain containing 2 | 116 | 116 | 88 | 98 | 104 | 1.4 | Group enr | 1 |
| SLC25A4 | solute carrier family 25 member 4 | 1532 | 1250 | 971 | 1256 | 1252 | 1.4 | Group enr | 1 |
| HSPB8 | heat shock 22kDa protein 8 | 378 | 302 | 254 | 254 | 297 | 1.3 | Enhanced | 1 |
| DIRAS1 | DIRAS family, GTP-binding RAS-like 1 | 36 | 36 | 58 | 56 | 46 | 1.3 | Group enr | 0 |
| PRSS42 | protease, serine, 42 | 5 | 4 | 0 | 0 | 3 | 1.3 | Group enr | 1 |
| ANGPTL7 | angiopoietin-like 7 | 3 | 4 | 13 | 3 | 6 | 1.3 | Enhanced | 0 |
| NEXN | nexilin | 353 | 433 | 199 | 299 | 321 | 1.3 | Enhanced | 1 |
| SMPX | small muscle protein, X-linked | 692 | 644 | 273 | 339 | 487 | 1.3 | Group enr | 0 |
| SYNDIG1 | synapse differentiation inducing 1 | 17 | 22 | 18 | 19 | 19 | 1.3 | Enhanced | 1 |
| PDE4DIP | phosphodiesterase 4D interacting protein | 3288 | 2066 | 702 | 1278 | 1834 | 1.3 | Group enr | 2 |
| SGSM1 | small G protein signaling modulator 1 | 3 | 1 | 23 | 14 | 10 | 1.3 | Enhanced | 1 |
| PPFIA4 | protein tyrosine phosphatase, receptor type, f polypeptide, interacting protein, alpha 4 | 24 | 26 | 17 | 13 | 20 | 1.2 | Enhanced | 2 |
| MYH7B | myosin, heavy chain 7B, cardiac muscle, beta | 72 | 42 | 33 | 34 | 45 | 1.2 | Group enr | 0 |
| C15orf56 | chromosome 15 open reading frame 56 | 1 | 1 | 2 | 3 | 2 | 1.2 | Enhanced | 1 |
| PALM2-AKAP2 | PALM2-AKAP2 readthrough | 23 | 24 | 24 | 28 | 25 | 1.2 | Enhanced | 1 |
| BVES | blood vessel epicardial substance | 20 | 32 | 11 | 27 | 23 | 1.2 | Enhanced | 2 |
| FAM155B | family with sequence similarity 155, member B | 1 | 3 | 18 | 28 | 13 | 1.2 | Group enr | 1 |
| TNNC1 | troponin C type 1 | 4999 | 3475 | 1722 | 2155 | 3088 | 1.2 | Group enr | 2 |
| FAM110B | family with sequence similarity 110, member B | 59 | 58 | 27 | 22 | 41 | 1.2 | Enhanced | 2 |
| MYH7 | myosin, heavy chain 7, cardiac muscle, beta | 3491 | 2764 | 446 | 509 | 1802 | 1.2 | Group enr | 2 |
| MYOM3 | myomesin 3 | 81 | 102 | 61 | 82 | 81 | 1.2 | Group enr | 2 |
| TRIM63 | tripartite motif containing 63, E3 ubiquitin protein ligase | 173 | 80 | 97 | 114 | 116 | 1.2 | Group enr | 0 |
| RNF103-CHMP3 | RNF103-CHMP3 readthrough | 2 | 4 | 3 | 3 | 3 | 1.2 | Enhanced | 1 |
| MYL2 | myosin, light chain 2, regulatory, cardiac, slow | 12037 | 8957 | 10 | 162 | 5291 | 1.1 | Group enr | 1 |
| ACTN2 | actinin, alpha 2 | 1209 | 1168 | 809 | 775 | 990 | 1.1 | Group enr | 1 |
| DTNA | dystrobrevin, alpha | 172 | 192 | 105 | 147 | 154 | 1.1 | Enhanced | 1 |
| FHL2 | four and a half LIM domains 2 | 430 | 1368 | 155 | 110 | 516 | 1.1 | Group enr | 3 |
| IGSF5 | immunoglobulin superfamily, member 5 | 4 | 2 | 3 | 2 | 3 | 1.1 | Enhanced | 1 |
| MYO18B | myosin XVIIIB | 103 | 54 | 51 | 44 | 63 | 1.1 | Group enr | 1 |
| ASB18 | ankyrin repeat and SOCS box containing 18 | 11 | 3 | 3 | 4 | 5 | 1.1 | Group enr | 0 |
| MASP1 | mannan-binding lectin serine peptidase 1 | 167 | 111 | 36 | 46 | 90 | 1.1 | Enhanced | 2 |
| SCHIP1 | schwannomin interacting protein 1 | 63 | 83 | 43 | 53 | 60 | 1.1 | Enhanced | 1 |
| ASPN | Aspirin | 77 | 112 | 46 | 60 | 74 | 1.1 | Enhanced | 2 |
| FBXO40 | F-box protein 40 | 48 | 58 | 81 | 82 | 67 | 1.1 | Group enr | 0 |
| SRL | sarcalumenin | 164 | 241 | 158 | 206 | 192 | 1.1 | Group enr | 2 |
| DUSP27 | dual specificity phosphatase 27 | 63 | 62 | 32 | 32 | 47 | 1.1 | Group enr | 1 |
| GABRA4 | gamma-aminobutyric acid A receptor, alpha 4 | 24 | 23 | 0 | 0 | 12 | 1.1 | Group enr | 1 |
| HSPB2 | Homo sapiens heat shock 27kDa protein 2, mRNA. | 95 | 49 | 50 | 99 | 73 | 1.0 | Enhanced | 2 |
| MYL3 | myosin, light chain 3, alkali; ventricular, skeletal, slow | 2354 | 1888 | 446 | 165 | 1213 | 1.0 | Group enr | 3 |
| C1QTNF9 | C1q and tumor necrosis factor related protein 9 | 3 | 6 | 2 | 2 | 3 | 1.0 | Enhanced | 1 |
| CDH13 | cadherin 13, H-cadherin | 74 | 190 | 91 | 87 | 110 | 1.0 | Enhanced | 2 |
| MYOM1 | myomesin 1 | 299 | 431 | 180 | 189 | 275 | 1.0 | Group enr | 2 |
| PLCL1 | phospholipase C-like 1 | 27 | 47 | 21 | 21 | 29 | 1.0 | Enhanced | 1 |
| UNC45B | unc-45 homolog B | 75 | 82 | 63 | 82 | 75 | 1.0 | Group enr | 1 |
| CYP2J2 | cytochrome P450, family 2, subfamily J, polypeptide 2 | 131 | 81 | 50 | 63 | 81 | 1.0 | Enhanced | 0 |
| ART3 | ADP-ribosyltransferase 3 | 43 | 27 | 46 | 28 | 36 | 1.0 | Group enr | 1 |
| B3GALT2 | UDP-Gal:betaGlcNAc beta 1,3-galactosyltransferase, polypeptide 2 | 14 | 16 | 19 | 24 | 18 | 0.9 | Enhanced | 1 |
| PPP1R14C | protein phosphatase 1, regulatory subunit 14C | 41 | 78 | 54 | 83 | 64 | 0.9 | Enhanced | 0 |
| SH3RF2 | SH3 domain containing ring finger 2 | 161 | 115 | 132 | 195 | 151 | 0.9 | Group enr | 1 |
| BCO2 | beta-carotene oxygenase 2 | 42 | 33 | 28 | 31 | 33 | 0.9 | Enhanced | 2 |
| CTAG2 | cancer/testis antigen 2 | 10 | 0 | 0 | 5 | 4 | 0.9 | Enhanced | 0 |
| AP001579.1 | Uncharacterized protein | 0 | 0 | 0 | 28 | 7 | 0.9 | Enhanced | 0 |
| PPM1J | protein phosphatase, Mg2+/Mn2+ dependent, 1J | 7 | 10 | 7 | 9 | 8 | 0.9 | Group enr | 1 |
| RP11-10A14.4 | Uncharacterized protein | 6 | 5 | 5 | 6 | 5 | 0.9 | Enhanced | 0 |
| GRIN2C | glutamate receptor, ionotropic, N-methyl D-aspartate 2C | 0 | 0 | 13 | 14 | 7 | 0.9 | Enhanced | 1 |
| KCNJ3 | potassium inwardly-rectifying channel, subfamily J, member 3 | 0 | 0 | 31 | 30 | 15 | 0.8 | Group enr | 1 |
| CSDC2 | cold shock domain containing C2, RNA binding | 65 | 58 | 29 | 38 | 48 | 0.8 | Enhanced | 1 |
| NDRG4 | NDRG family member 4 | 294 | 344 | 111 | 135 | 221 | 0.8 | Enhanced | 2 |
| FRMD5 | FERM domain containing 5 | 13 | 7 | 10 | 9 | 10 | 0.8 | Group enr | 2 |
| RASL11B | RAS-like, family 11, member B | 30 | 17 | 29 | 26 | 25 | 0.8 | Enhanced | 1 |
| GATA4 | GATA binding protein 4 | 75 | 51 | 74 | 71 | 68 | 0.8 | Enhanced | 1 |
| C14orf180 | chromosome 14 open reading frame 180 | 45 | 52 | 47 | 82 | 56 | 0.7 | Group enr | 1 |
| TMEM178B | transmembrane protein 178B | 2 | 6 | 9 | 16 | 9 | 0.7 | Enhanced | 1 |
| SLC27A6 | solute carrier family 27, member 6 | 16 | 15 | 17 | 27 | 18 | 0.7 | Enhanced | 1 |
| ERBB4 | v-erb-b2 avian erythroblastic leukemia viral oncogene homolog 4 | 3 | 5 | 5 | 4 | 4 | 0.7 | Group enr | 3 |
| RBFOX1 | RNA binding protein, fox-1 homolog 1 | 11 | 27 | 20 | 25 | 21 | 0.7 | Group enr | 1 |
| EBF2 | early B-cell factor 2 | 6 | 12 | 9 | 8 | 8 | 0.7 | Enhanced | 1 |
| GHRH | growth hormone releasing hormone | 0 | 0 | 5 | 2 | 2 | 0.7 | Enhanced | 0 |
| GLP1R | glucagon-like peptide 1 receptor | 2 | 0 | 2 | 4 | 2 | 0.7 | Enhanced | 0 |
| P2RX3 | purinergic receptor P2X, ligand-gated ion channel, 3 | 3 | 4 | 0 | 4 | 3 | 0.7 | Enhanced | 1 |
| C20orf26 | chromosome 20 open reading frame 26 | 30 | 26 | 1 | 1 | 14 | 0.7 | Group enr | 0 |
| ADAM23 | ADAM metallopeptidase domain 23 | 16 | 33 | 15 | 14 | 19 | 0.7 | Enhanced | 2 |
| FGF1 | fibroblast growth factor 1 | 58 | 69 | 42 | 58 | 57 | 0.7 | Enhanced | 2 |
| TCF15 | transcription factor 15 | 5 | 2 | 2 | 5 | 4 | 0.7 | Group enr | 1 |
| FAM216B | family with sequence similarity 216, member B | 0 | 0 | 12 | 19 | 8 | 0.6 | Group enr | 1 |
| KCNJ4 | potassium inwardly-rectifying channel, subfamily J, member 4 | 19 | 12 | 10 | 9 | 12 | 0.6 | Group enr | 1 |
| THBS4 | thrombospondin 4 | 352 | 242 | 8 | 37 | 160 | 0.6 | Group enr | 1 |
| ATP1A3 | ATPase, Na+/K+ transporting, alpha 3 polypeptide | 77 | 81 | 141 | 114 | 103 | 0.6 | Group enr | 3 |
| GUCA1C | guanylate cyclase activator 1C | 36 | 6 | 0 | 0 | 10 | 0.6 | Group enr | 1 |
| PPP1R1C | protein phosphatase 1, regulatory subunit 1C | 55 | 45 | 14 | 19 | 33 | 0.5 | Group enr | 1 |
| PAQR9 | progestin and adipoQ receptor family member IX | 8 | 8 | 6 | 7 | 7 | 0.5 | Enhanced | 1 |
| ADAM11 | ADAM metallopeptidase domain 11 | 0 | 3 | 9 | 17 | 7 | 0.5 | Enhanced | 0 |
| SAMD4A | sterile alpha motif domain containing 4A | 44 | 53 | 41 | 44 | 45 | 0.5 | Group enr | 1 |
| IRX6 | iroquois homeobox 6 | 15 | 5 | 0 | 0 | 5 | 0.5 | Enhanced | 1 |
| GRM1 | glutamate receptor, metabotropic 1 | 6 | 3 | 0 | 0 | 2 | 0.5 | Group enr | 1 |
| CAMK2B | calcium/calmodulin-dependent protein kinase II beta | 34 | 46 | 48 | 61 | 47 | 0.5 | Group enr | 5 |
| CLGN | Calmegin | 24 | 40 | 44 | 56 | 41 | 0.5 | Group enr | 1 |
| FBXL22 | F-box and leucine-rich repeat protein 22 | 10 | 13 | 14 | 9 | 12 | 0.5 | Group enr | 2 |
| HCN2 | hyperpolarization activated cyclic nucleotide-gated potassium channel 2 | 12 | 2 | 9 | 7 | 8 | 0.5 | Group enr | 0 |
| ST8SIA5 | ST8 alpha-N-acetyl-neuraminide alpha-2,8-sialyltransferase 5 | 5 | 4 | 4 | 4 | 4 | 0.5 | Group enr | 2 |
| KCNIP2 | Kv channel interacting protein 2 | 4 | 16 | 271 | 217 | 127 | 0.5 | Group enr | 0 |
| SCN2B | sodium channel, voltage-gated, type II, beta subunit | 12 | 12 | 4 | 5 | 8 | 0.4 | Group enr | 1 |
| ESRRB | estrogen-related receptor beta | 3 | 3 | 4 | 5 | 4 | 0.4 | Enhanced | 0 |
| HCN4 | hyperpolarization activated cyclic nucleotide-gated potassium channel 4 | 1 | 2 | 4 | 1 | 2 | 0.4 | Enhanced | 0 |
| MLF1 | myeloid leukemia factor 1 | 70 | 120 | 150 | 133 | 118 | 0.4 | Group enr | 1 |
| SHD | Src homology 2 domain containing transforming protein D | 0 | 0 | 14 | 17 | 8 | 0.4 | Group enr | 1 |
| ALDOC | aldolase C, fructose-bisphosphate | 106 | 187 | 103 | 102 | 125 | 0.4 | Group enr | 2 |
| TYRP1 | tyrosinase-related protein 1 | 65 | 74 | 20 | 35 | 49 | 0.4 | Group enr | 2 |
| FAM69C | family with sequence similarity 69, member C | 11 | 7 | 1 | 3 | 5 | 0.4 | Group enr | 1 |
| AC120194.1 | N/A | 20 | 0 | 0 | 0 | 5 | 0.3 | Group enr | 0 |
| LPL | lipoprotein lipase | 448 | 425 | 29 | 81 | 246 | 0.3 | Group enr | 0 |
| DCLK2 | doublecortin-like kinase 2 | 23 | 16 | 6 | 7 | 13 | 0.3 | Group enr | 1 |
| DNAAF3 | dynein, axonemal, assembly factor 3 | 38 | 16 | 5 | 4 | 16 | 0.2 | Group enr | 1 |
| GABRB1 | gamma-aminobutyric acid (GABA) A receptor, beta 1 | 6 | 8 | 1 | 2 | 4 | 0.2 | Group enr | 1 |
| SYNPR | Synaptoporin | 1 | 0 | 16 | 31 | 12 | 0.2 | Group enr | 2 |
| LMAN1L | lectin, mannose-binding, 1 like | 0 | 1 | 36 | 26 | 16 | 0.2 | Group enr | 1 |

^1^ RNA category of cardiac muscle elevated expression defined as enriched, group enriched (group enr) or enhanced.
