# Supplementary material for: The human cardiac and skeletal muscle proteomes defined by transcriptomics and antibody-based profiling
Source: BMC Genomics. 2015 Jun 25;16(1):475. doi: 10.1186/s12864-015-1686-y (PMC4479346; doi:10.1186/s12864-015-1686-y)
Supplement: Additional file 2: Table S2. — List of 47 genes elevated in cardiac muscle and differentially expressed between ventricle and auricle samples. The list includes genes showing at least a five-fold difference between mean FPKM values of ventricle and auricle samples, respectively. [file 12864_2015_1686_MOESM2_ESM.docx]

**Supplemental Table 2. List of 47 genes elevated in cardiac muscle and differentially expressed between ventricle and auricle samples.** The list includes genes showing at least a five-fold difference between mean FPKM values of ventricle and auricle samples, respectively.

| **Gene** | **Description** | **Mean mRNA level ventricle (FPKM)** | **Mean mRNA level auricle**  **(FPKM)** | **Fold-difference ventricle/auricle** |  |
| --- | --- | --- | --- | --- | --- |
| HAND1 | heart and neural crest derivatives expressed 1 | 11 | 2 | 5 |  |
| DNAAF3 | dynein, axonemal, assembly factor 3 | 27 | 5 | 6 |  |
| MYH7 | myosin, heavy chain 7, cardiac muscle, beta | 3128 | 477 | 7 |  |
| FHL2 | four and a half LIM domains 2 | 899 | 132 | 7 |  |
| MYL3 | myosin, light chain 3, alkali; ventricular, skeletal, slow | 2121 | 306 | 7 |  |
| KBTBD12 | kelch repeat and BTB domain containing 12 | 19 | 3 | 8 |  |
| LPL | lipoprotein lipase | 437 | 55 | 8 |  |
| CRIP3 | cysteine-rich protein 3 | 36 | 4 | 8 |  |
| ANKRD2 | ankyrin repeat domain 2 | 65 | 7 | 9 |  |
| XIRP2 | xin actin-binding repeat containing 2 | 175 | 14 | 13 |  |
| THBS4 | thrombospondin 4 | 297 | 23 | 13 |  |
| PRSS42 | protease, serine, 42 | 5 | 0.3 | 18 |  |
| ST8SIA2 | ST8 alpha-N-acetyl-neuraminide alpha-2.8-sialyltransferase 2 | 6 | 0.3 | 18 |  |
| C20orf26 | chromosome 20 open reading frame 26 | 28 | 1 | 24 |  |
| FGF18 | fibroblast growth factor 18 | 13 | 0.6 | 24 |  |
| GABRA4 | gamma-aminobutyric acid A receptor. alpha 4 | 23 | 0.3 | 70 |  |
| GRM1 | glutamate receptor. metabotropic 1 | 4 | 0.1 | 74 |  |
| IRX6 | iroquois homeobox 6 | 10 | 0.1 | 97 |  |
| MYL2 | myosin. light chain 2. regulatory. cardiac. slow | 10497 | 86 | 122 |  |
| AC120194.1 | N/A | 10 | <0.1 | 102 |  |
| GUCA1C | guanylate cyclase activator 1C | 21 | <0.1 | 210 |  |
| ADAM11 | ADAM metallopeptidase domain 11 | 2 | 13 | 0.2 |  |
| CORIN | corin. serine peptidase | 16 | 111 | 0.1 |  |
| NPPA | natriuretic peptide A | 1563 | 11824 | 0.1 |  |
| SGSM1 | small G protein signaling modulator 1 | 2 | 18 | 0.1 |  |
| KCNA5 | potassium voltage-gated channel. shaker-related subfamily. member 5 | 3 | 32 | 0.1 |  |
| FAM155B | family with sequence similarity 155. member B | 2 | 23 | 0.1 |  |
| MYL4 | myosin. light chain 4. alkali; atrial. embryonic | 106 | 1846 | 0.1 |  |
| DHRS7C | dehydrogenase/reductase member 7C | 7 | 125 | 0.1 |  |
| GSG1L | GSG1-like | 1 | 20 | 0.1 |  |
| KCNIP2 | Kv channel interacting protein 2 | 10 | 244 | <0.1 |  |
| MYH6 | myosin. heavy chain 6. cardiac muscle. alpha | 56 | 1511 | <0.1 |  |
| SYNPR | synaptoporin | 1 | 23 | <0.1 |  |
| SGK110 | putative uncharacterized serine/threonine-protein kinase SgK110 | 1 | 30 | <0.1 |  |
| GHRH | growth hormone releasing hormone | <0.1 | 4 | <0.1 |  |
| LMAN1L | lectin. mannose-binding. 1 like | 1 | 31 | <0.1 |  |
| GRIN2C | glutamate receptor. ionotropic. N-methyl D-aspartate 2C | 0.2 | 14 | <0.1 |  |
| DCAF8 | DDB1- and CUL4-associated factor 8 | 0.1 | 5 | <0.1 |  |
| AP001579.1 | Uncharacterized protein | <0.1 | 14 | <0.1 |  |
| FAM216B | family with sequence similarity 216. member B | 0.1 | 15 | <0.1 |  |
| CHRNE | cholinergic receptor. nicotinic. epsilon | 0.3 | 74 | <0.1 |  |
| SBK2 | SH3-binding domain kinase family. member 2 | 0.1 | 40 | <0.1 |  |
| SHD | Src homology 2 domain containing transforming protein D | 0.0 | 16 | <0.1 |  |
| MYBPHL | myosin binding protein H-like | 0.3 | 177 | <0.1 |  |
| KCNJ3 | potassium inwardly-rectifying channel. subfamily J. member 3 | 0.1 | 30 | <0.1 |  |
| MYBPC3 | myosin binding protein C. cardiac | <0.1 | 256 | <0.1 |  |
| BMP10 | bone morphogenetic protein 10 | <0.1 | 605 | <0.1 |  |

.
