# Supplementary material for: The human cardiac and skeletal muscle proteomes defined by transcriptomics and antibody-based profiling
Source: BMC Genomics. 2015 Jun 25;16(1):475. doi: 10.1186/s12864-015-1686-y (PMC4479346; doi:10.1186/s12864-015-1686-y)
Supplement: Additional file 3: Table S3. — List of the 371 genes elevated in skeletal muscle. The list is sorted after tissue-specific score which is the FPKM value in skeletal muscle divided by the maximum FPKM in all other tissues. [file 12864_2015_1686_MOESM3_ESM.docx]

**Supplemental Table 3. List of the 371 genes elevated in skeletal muscle.** The list is sorted after tissue-specific score which is the FPKM value in skeletal muscle divided by the maximum FPKM in all other tissues.

| **Gene** | **Description** | **mRNA level (FPKM) Sample 1** | **mRNA level (FPKM) Sample 2** | **mRNA level (FPKM) Sample 3** | **mRNA level (FPKM) Sample 4** | **mRNA level (FPKM) Sample 5** | **Mean mRNA level (FPKM)** | **Tissue-specific score** | **Category** | **Number of antibodies with tissue data at www.proteinatlas.org** |
| --- | --- | --- | --- | --- | --- | --- | --- | --- | --- | --- |
| MYH1 | myosin, heavy chain 1, skeletal muscle, adult | 1678 | 1002 | 1535 | 709 | 875 | 1160 | 321.1 | Enriched | 2 |
| IDI2 | isopentenyl-diphosphate delta isomerase 2 | 60 | 85 | 66 | 112 | 112 | 87 | 294.7 | Enriched | 1 |
| MYBPC2 | myosin binding protein C, fast type | 1355 | 730 | 1195 | 981 | 1409 | 1134 | 87.3 | Enriched | 2 |
| LBX1 | ladybird homeobox 1 | 10 | 6 | 7 | 9 | 8 | 8 | 80.1 | Enriched | 1 |
| ATP2A1 | ATPase, Ca++ transporting, cardiac muscle, fast twitch 1 | 2651 | 1466 | 1664 | 1542 | 2357 | 1936 | 74.4 | Enriched | 1 |
| NEB | Nebulin | 2801 | 2826 | 2411 | 2236 | 1130 | 2281 | 62.5 | Enriched | 1 |
| KCNA7 | potassium voltage-gated channel, shaker-related subfamily, member 7 | 35 | 26 | 30 | 29 | 31 | 30 | 57.8 | Enriched | 0 |
| MYLK2 | myosin light chain kinase 2 | 204 | 167 | 262 | 237 | 447 | 263 | 50.1 | Enriched | 2 |
| C8orf22 | chromosome 8 open reading frame 22 | 87 | 338 | 390 | 415 | 619 | 370 | 49.0 | Enriched | 1 |
| MYH2 | myosin, heavy chain 2, skeletal muscle, adult | 2411 | 1615 | 1132 | 1842 | 2326 | 1865 | 47.9 | Enriched | 2 |
| MYLPF | myosin light chain, phosphorylatable, fast skeletal muscle | 3738 | 2706 | 2790 | 3169 | 3962 | 3273 | 47.8 | Enriched | 1 |
| MYH4 | myosin, heavy chain 4, skeletal muscle | 9 | 5 | 5 | 6 | 10 | 7 | 47.1 | Enriched | 1 |
| TNNT3 | troponin T type 3 | 5135 | 3292 | 3547 | 3929 | 3984 | 3977 | 45.6 | Enriched | 2 |
| TNNC2 | troponin C type 2 | 5715 | 3630 | 3943 | 4380 | 5100 | 4554 | 43.7 | Enriched | 1 |
| YIPF7 | Yip1 domain family, member 7 | 28 | 30 | 35 | 32 | 37 | 32 | 41.3 | Enriched | 1 |
| CACNA1S | calcium channel, voltage-dependent, L type, alpha 1S subunit | 103 | 86 | 104 | 109 | 127 | 106 | 36.2 | Enriched | 3 |
| C20orf166 | chromosome 20 open reading frame 166 | 195 | 192 | 337 | 260 | 127 | 222 | 35.4 | Enriched | 0 |
| ANKRD23 | ankyrin repeat domain 23 | 465 | 415 | 415 | 568 | 546 | 482 | 33.4 | Enriched | 1 |
| FGF6 | fibroblast growth factor 6 | 7 | 4 | 10 | 6 | 6 | 6 | 31.6 | Enriched | 0 |
| JSRP1 | junctional sarcoplasmic reticulum protein 1 | 96 | 103 | 119 | 116 | 155 | 118 | 30.9 | Enriched | 1 |
| PPP1R27 | protein phosphatase 1, regulatory subunit 27 | 211 | 92 | 179 | 134 | 234 | 170 | 30.9 | Enriched | 1 |
| CASQ1 | calsequestrin 1 | 1330 | 1074 | 1165 | 1140 | 1635 | 1269 | 29.9 | Enriched | 3 |
| RP11-766F14.2 | Protein LOC285556 | 32 | 23 | 20 | 17 | 55 | 29 | 29.6 | Enriched | 0 |
| CACNG1 | calcium channel, voltage-dependent, gamma subunit 1 | 133 | 111 | 129 | 132 | 136 | 128 | 29.5 | Enriched | 1 |
| SMTNL1 | smoothelin-like 1 | 242 | 169 | 123 | 210 | 391 | 227 | 29.4 | Enriched | 1 |
| MYOZ1 | myozenin 1 | 1467 | 1416 | 1451 | 1429 | 1653 | 1483 | 29.1 | Enriched | 2 |
| TNNI2 | troponin I type 2 | 3636 | 2178 | 2383 | 2814 | 3152 | 2833 | 27.9 | Enriched | 2 |
| ASB12 | ankyrin repeat and SOCS box containing 12 | 38 | 27 | 37 | 27 | 45 | 35 | 27.7 | Enriched | 1 |
| DCD | Dermcidin | 0 | 0 | 0 | 0 | 19 | 4 | 26.8 | Enriched | 0 |
| PYGM | phosphorylase, glycogen, muscle | 2128 | 1886 | 1959 | 1839 | 2080 | 1979 | 26.4 | Enriched | 1 |
| UCP3 | uncoupling protein 3 | 98 | 51 | 57 | 69 | 142 | 84 | 26.2 | Enriched | 0 |
| RYR1 | ryanodine receptor 1 | 409 | 372 | 409 | 430 | 516 | 427 | 23.6 | Enriched | 1 |
| DUPD1 | dual specificity phosphatase and pro isomerase domain containing 1 | 41 | 27 | 35 | 41 | 51 | 39 | 22.5 | Enriched | 1 |
| PRKAG3 | protein kinase, AMP-activated, gamma 3 non-catalytic subunit | 67 | 24 | 41 | 20 | 37 | 38 | 21.3 | Enriched | 0 |
| ART1 | ADP-ribosyltransferase 1 | 16 | 23 | 18 | 40 | 35 | 27 | 21.2 | Enriched | 1 |
| CLCN1 | chloride channel, voltage-sensitive 1 | 26 | 28 | 20 | 32 | 18 | 25 | 21.1 | Enriched | 0 |
| CACNG6 | calcium channel, voltage-dependent, gamma subunit 6 | 37 | 25 | 29 | 29 | 51 | 34 | 20.8 | Enriched | 0 |
| TMOD4 | tropomodulin 4 | 307 | 331 | 374 | 372 | 380 | 353 | 20.6 | Enriched | 1 |
| AC025262.1 | Mesenchymal stem cell protein DSC96; Uncharacterized protein | 0 | 10 | 0 | 0 | 0 | 2 | 19.9 | Enriched | 0 |
| CHRNG | cholinergic receptor, nicotinic, gamma | 9 | 11 | 13 | 14 | 4 | 10 | 19.6 | Enriched | 0 |
| STAC3 | SH3 and cysteine rich domain 3 | 861 | 866 | 829 | 833 | 810 | 840 | 19.4 | Enriched | 0 |
| JPH1 | junctophilin 1 | 93 | 105 | 109 | 101 | 109 | 103 | 18.5 | Enriched | 3 |
| MYOT | Myotilin | 634 | 818 | 841 | 888 | 473 | 731 | 18.2 | Enriched | 2 |
| GADL1 | glutamate decarboxylase-like 1 | 11 | 10 | 11 | 13 | 8 | 11 | 18.1 | Enriched | 2 |
| MYADML2 | myeloid-associated differentiation marker-like 2 | 38 | 22 | 34 | 31 | 52 | 35 | 18.0 | Enriched | 2 |
| MYBPC1 | myosin binding protein C, slow type | 2747 | 2520 | 2767 | 2924 | 2602 | 2712 | 17.5 | Enriched | 2 |
| CHRNA10 | cholinergic receptor, nicotinic, alpha 10 | 8 | 18 | 13 | 19 | 20 | 16 | 17.4 | Enriched | 1 |
| MYL1 | myosin, light chain 1, alkali; skeletal, fast | 4325 | 3697 | 3774 | 4501 | 4677 | 4195 | 17.1 | Enriched | 1 |
| FEM1A | fem-1 homolog a | 64 | 109 | 98 | 122 | 163 | 111 | 16.8 | Enriched | 1 |
| KLHL41 | kelch-like family member 41 | 1492 | 1590 | 1335 | 1552 | 1342 | 1462 | 16.3 | Enriched | 3 |
| TNNI1 | troponin I type 1 | 1024 | 2193 | 1728 | 1796 | 1812 | 1711 | 16.2 | Enriched | 2 |
| SYPL2 | synaptophysin-like 2 | 159 | 133 | 165 | 119 | 139 | 143 | 16.1 | Enriched | 1 |
| CHRND | cholinergic receptor, nicotinic, delta | 19 | 11 | 14 | 7 | 28 | 16 | 15.8 | Enriched | 0 |
| KLHL33 | kelch-like family member 33 | 17 | 31 | 24 | 27 | 33 | 26 | 15.7 | Enriched | 1 |
| TRIM72 | tripartite motif containing 72 | 36 | 36 | 40 | 43 | 49 | 41 | 14.9 | Enriched | 1 |
| FHL3 | four and a half LIM domains 3 | 446 | 582 | 615 | 623 | 667 | 587 | 14.3 | Enriched | 1 |
| AMPD1 | adenosine monophosphate deaminase 1 | 231 | 218 | 179 | 206 | 196 | 206 | 13.7 | Enriched | 2 |
| CDH15 | cadherin 15, type 1, M-cadherin | 17 | 15 | 18 | 18 | 20 | 17 | 13.5 | Enriched | 1 |
| TMEM38A | transmembrane protein 38A | 273 | 185 | 191 | 184 | 237 | 214 | 13.4 | Enriched | 2 |
| RAPSN | receptor-associated protein of the synapse | 29 | 34 | 33 | 33 | 36 | 33 | 13.4 | Enriched | 1 |
| CA3 | carbonic anhydrase III, muscle specific | 1033 | 1301 | 1898 | 1476 | 1394 | 1420 | 13.2 | Enriched | 3 |
| MYF6 | myogenic factor 6 | 70 | 89 | 64 | 87 | 194 | 101 | 12.9 | Enriched | 0 |
| LRRC30 | leucine rich repeat containing 30 | 5 | 5 | 4 | 8 | 8 | 6 | 12.8 | Enriched | 1 |
| VGLL2 | vestigial like 2 | 44 | 43 | 54 | 55 | 67 | 52 | 12.7 | Enriched | 0 |
| MSS51 | MSS51 mitochondrial translational activator | 130 | 62 | 115 | 89 | 112 | 102 | 12.2 | Enriched | 1 |
| ASB16 | ankyrin repeat and SOCS box containing 16 | 20 | 17 | 23 | 26 | 43 | 26 | 12.2 | Enriched | 0 |
| MYOZ3 | myozenin 3 | 105 | 95 | 95 | 116 | 122 | 107 | 12.1 | Enriched | 1 |
| KLHL34 | kelch-like family member 34 | 6 | 10 | 8 | 9 | 12 | 9 | 11.6 | Enriched | 1 |
| KLHL40 | kelch-like family member 40 | 48 | 45 | 51 | 77 | 87 | 62 | 11.6 | Enriched | 2 |
| CALML6 | calmodulin-like 6 | 24 | 75 | 49 | 69 | 23 | 48 | 10.9 | Enriched | 1 |
| LSMEM1 | leucine-rich single-pass membrane protein 1 | 41 | 36 | 43 | 40 | 46 | 41 | 10.4 | Enriched | 1 |
| SLN | Sarcolipin | 1588 | 1497 | 1428 | 1935 | 2509 | 1791 | 10.4 | Enriched | 1 |
| MUSTN1 | musculoskeletal, embryonic nuclear protein 1 | 337 | 326 | 313 | 435 | 650 | 412 | 10.4 | Enriched | 0 |
| ATP1B4 | ATPase, Na+/K+ transporting, beta 4 polypeptide | 19 | 21 | 31 | 32 | 14 | 23 | 10.3 | Enriched | 1 |
| AC106722.1 | HCG1806964, isoform CRA_a; Uncharacterized protein; cDNA FLJ55426; cDNA, FLJ79414 | 36 | 26 | 21 | 47 | 65 | 39 | 10.1 | Enriched | 0 |
| PHKG1 | phosphorylase kinase, gamma 1 | 161 | 127 | 118 | 149 | 177 | 146 | 9.9 | Enriched | 1 |
| ENO3 | enolase 3 | 4247 | 3204 | 3592 | 2967 | 3849 | 3572 | 9.3 | Enriched | 2 |
| TNNT1 | troponin T type 1 | 3277 | 3796 | 3618 | 4146 | 3135 | 3595 | 9.1 | Enriched | 1 |
| PLCD4 | phospholipase C, delta 4 | 69 | 58 | 76 | 66 | 54 | 64 | 9.1 | Enriched | 3 |
| FBP2 | fructose-1,6-bisphosphatase 2 | 331 | 299 | 227 | 136 | 302 | 259 | 9.0 | Enriched | 1 |
| ASB10 | ankyrin repeat and SOCS box containing 10 | 32 | 36 | 33 | 34 | 36 | 34 | 8.9 | Enriched | 1 |
| MYOG | myogenin | 20 | 46 | 30 | 32 | 30 | 32 | 8.2 | Enriched | 1 |
| RGS9BP | regulator of G protein signaling 9 binding protein | 5 | 2 | 3 | 3 | 5 | 3 | 8.1 | Enriched | 1 |
| MAPK12 | mitogen-activated protein kinase 12 | 93 | 93 | 112 | 92 | 137 | 105 | 8.0 | Enriched | 2 |
| MYOD1 | myogenic differentiation 1 | 9 | 9 | 13 | 12 | 7 | 10 | 7.8 | Enriched | 2 |
| BIN1 | bridging integrator 1 | 657 | 469 | 627 | 519 | 869 | 628 | 7.8 | Enriched | 3 |
| RXRG | retinoid X receptor, gamma | 20 | 34 | 60 | 24 | 55 | 39 | 7.7 | Enriched | 1 |
| RTN2 | reticulon 2 | 191 | 154 | 167 | 186 | 236 | 187 | 7.3 | Enriched | 1 |
| TPM2 | tropomyosin 2 | 6412 | 4724 | 5026 | 5504 | 6068 | 5547 | 7.1 | Enriched | 0 |
| CKM | creatine kinase, muscle | 9008 | 7724 | 7744 | 7822 | 7832 | 8026 | 6.8 | Enriched | 1 |
| NOS1 | nitric oxide synthase 1 | 11 | 18 | 21 | 13 | 17 | 16 | 6.3 | Enriched | 1 |
| SLC29A2 | solute carrier family 29, member 2 | 37 | 51 | 51 | 57 | 28 | 45 | 6.2 | Enriched | 1 |
| DDIT4L | DNA-damage-inducible transcript 4-like | 92 | 106 | 186 | 56 | 163 | 120 | 6.2 | Enriched | 1 |
| CACNB1 | calcium channel, voltage-dependent, beta 1 subunit | 223 | 164 | 195 | 158 | 203 | 189 | 6.1 | Enriched | 2 |
| ACTA1 | actin, alpha 1, skeletal muscle | 12862 | 10931 | 10414 | 10987 | 11875 | 11414 | 6.1 | Enriched | 2 |
| OBSCN | obscurin, cytoskeletal calmodulin and titin-interacting RhoGEF | 261 | 272 | 314 | 305 | 351 | 301 | 6.0 | Enriched | 2 |
| KCNJ12 | potassium inwardly-rectifying channel, subfamily J, member 12 | 24 | 22 | 26 | 24 | 37 | 27 | 6.0 | Enriched | 1 |
| PRSS56 | protease, serine, 56 | 3 | 3 | 3 | 3 | 5 | 3 | 5.9 | Enriched | 0 |
| MN1 | meningioma 1 | 22 | 28 | 27 | 25 | 28 | 26 | 5.8 | Enriched | 1 |
| KCNJ11 | potassium inwardly-rectifying channel, subfamily J, member 11 | 30 | 36 | 34 | 22 | 32 | 31 | 5.7 | Enriched | 1 |
| ACHE | acetylcholinesterase | 44 | 54 | 65 | 70 | 82 | 63 | 5.6 | Enriched | 1 |
| DUSP13 | dual specificity phosphatase 13 | 198 | 143 | 161 | 152 | 243 | 179 | 5.6 | Enriched | 0 |
| SEMA6C | sema domain, transmembrane domain, and cytoplasmic domain, 6C | 129 | 125 | 115 | 141 | 142 | 130 | 5.5 | Enriched | 0 |
| PITX3 | paired-like homeodomain 3 | 5 | 8 | 4 | 9 | 5 | 6 | 5.4 | Enriched | 1 |
| ASB14 | ankyrin repeat and SOCS box containing 14 | 24 | 27 | 11 | 18 | 12 | 18 | 5.4 | Enriched | 0 |
| SMTNL2 | smoothelin-like 2 | 211 | 112 | 130 | 95 | 166 | 143 | 5.2 | Enriched | 1 |
| TPM3 | tropomyosin 3 | 1113 | 1578 | 1530 | 1601 | 1392 | 1443 | 5.1 | Enriched | 2 |
| KBTBD13 | kelch repeat and BTB domain containing 13 | 12 | 10 | 11 | 13 | 19 | 13 | 5.1 | Enriched | 1 |
| ADSSL1 | adenylosuccinate synthase like 1 | 386 | 401 | 451 | 411 | 554 | 441 | 5.1 | Enriched | 0 |
| PROB1 | proline-rich basic protein 1 | 17 | 20 | 19 | 21 | 30 | 22 | 5.0 | Enriched | 1 |
| MAFA | v-maf avian musculoaponeurotic fibrosarcoma oncogene homolog A | 13 | 6 | 7 | 6 | 13 | 9 | 4.9 | Group enr | 0 |
| LRRC20 | leucine rich repeat containing 20 | 134 | 128 | 130 | 150 | 195 | 148 | 4.8 | Group enr | 1 |
| TTN | titin | 251 | 343 | 254 | 283 | 212 | 269 | 4.8 | Group enr | 2 |
| PLN | phospholamban | 229 | 313 | 254 | 357 | 150 | 261 | 4.7 | Group enr | 2 |
| ANK1 | ankyrin 1, erythrocytic | 322 | 320 | 348 | 303 | 302 | 319 | 4.7 | Enhanced | 3 |
| PIGY | phosphatidylinositol glycan anchor biosynthesis, class Y | 0 | 5 | 0 | 1 | 1 | 1 | 4.7 | Enhanced | 1 |
| FABP3 | fatty acid binding protein 3, muscle and heart | 348 | 385 | 552 | 590 | 384 | 452 | 4.6 | Group enr | 2 |
| RPL3L | ribosomal protein L3-like | 273 | 297 | 325 | 292 | 292 | 296 | 4.6 | Group enr | 1 |
| ASB2 | ankyrin repeat and SOCS box containing 2 | 197 | 139 | 211 | 127 | 230 | 181 | 4.5 | Group enr | 1 |
| AC117395.1 | LOC646903 protein; Uncharacterized protein | 11 | 9 | 11 | 14 | 19 | 13 | 4.5 | Enhanced | 0 |
| CMYA5 | cardiomyopathy associated 5 | 714 | 871 | 882 | 581 | 381 | 686 | 4.4 | Group enr | 0 |
| ABRA | actin-binding Rho activating protein | 83 | 30 | 50 | 80 | 42 | 57 | 4.3 | Group enr | 1 |
| PGPEP1L | pyroglutamyl-peptidase I-like | 12 | 8 | 6 | 12 | 13 | 10 | 4.3 | Enhanced | 1 |
| PDLIM3 | PDZ and LIM domain 3 | 976 | 792 | 788 | 1160 | 1347 | 1013 | 4.3 | Enhanced | 1 |
| CAV3 | caveolin 3 | 63 | 76 | 76 | 61 | 79 | 71 | 4.2 | Group enr | 2 |
| DUSP26 | dual specificity phosphatase 26 | 129 | 113 | 105 | 142 | 203 | 138 | 4.1 | Enhanced | 1 |
| NEURL | neuralized homolog | 41 | 42 | 57 | 55 | 79 | 55 | 4.1 | Enhanced | 0 |
| TBX1 | T-box 1 | 20 | 21 | 26 | 20 | 19 | 21 | 4.1 | Enhanced | 1 |
| KLHL30 | kelch-like family member 30 | 19 | 22 | 28 | 20 | 33 | 24 | 4.0 | Enhanced | 1 |
| LINC00346 | long intergenic non-protein coding RNA 346 | 9 | 10 | 9 | 8 | 16 | 10 | 3.9 | Enhanced | 0 |
| FNDC5 | fibronectin type III domain containing 5 | 101 | 138 | 146 | 230 | 136 | 150 | 3.9 | Enhanced | 1 |
| MLIP | muscular LMNA-interacting protein | 41 | 56 | 56 | 49 | 45 | 49 | 3.9 | Group enr | 1 |
| NT5C1A | 5'-nucleotidase, cytosolic IA | 34 | 26 | 20 | 21 | 34 | 27 | 3.8 | Group enr | 2 |
| ASB5 | ankyrin repeat and SOCS box containing 5 | 78 | 84 | 96 | 95 | 82 | 87 | 3.8 | Enhanced | 1 |
| TEAD4 | TEA domain family member 4 | 48 | 29 | 30 | 32 | 64 | 41 | 3.8 | Enhanced | 1 |
| MYLK3 | myosin light chain kinase 3 | 10 | 34 | 20 | 22 | 17 | 21 | 3.7 | Group enr | 1 |
| MYLK4 | myosin light chain kinase family, member 4 | 25 | 6 | 6 | 12 | 10 | 12 | 3.7 | Group enr | 1 |
| LRRC14B | leucine rich repeat containing 14B | 27 | 28 | 30 | 25 | 43 | 30 | 3.7 | Group enr | 2 |
| MYF5 | myogenic factor 5 | 2 | 2 | 2 | 3 | 3 | 2 | 3.7 | Enhanced | 1 |
| C1orf170 | chromosome 1 open reading frame 170 | 72 | 80 | 89 | 80 | 85 | 81 | 3.7 | Enhanced | 0 |
| PHKA1 | phosphorylase kinase, alpha 1 | 53 | 34 | 32 | 30 | 36 | 37 | 3.6 | Enhanced | 1 |
| MSTN | myostatin | 7 | 2 | 6 | 3 | 5 | 5 | 3.6 | Enhanced | 1 |
| SNAI3 | snail family zinc finger 3 | 10 | 13 | 9 | 19 | 9 | 12 | 3.6 | Enhanced | 1 |
| IGFN1 | immunoglobulin-like and fibronectin type III domain containing 1 | 233 | 3 | 19 | 1 | 41 | 59 | 3.5 | Group enr | 0 |
| EN1 | engrailed homeobox 1 | 6 | 4 | 6 | 4 | 8 | 5 | 3.4 | Group enr | 1 |
| C10orf71 | chromosome 10 open reading frame 71 | 81 | 110 | 121 | 128 | 148 | 118 | 3.3 | Group enr | 1 |
| PRKCQ | protein kinase C, theta | 37 | 53 | 40 | 48 | 51 | 46 | 3.3 | Enhanced | 0 |
| TRIM54 | tripartite motif containing 54 | 271 | 252 | 282 | 232 | 264 | 260 | 3.3 | Group enr | 1 |
| CHRNA1 | cholinergic receptor, nicotinic, alpha 1 | 7 | 9 | 9 | 9 | 13 | 9 | 3.3 | Group enr | 1 |
| HSPB3 | heat shock 27kDa protein 3 | 63 | 87 | 72 | 72 | 84 | 76 | 3.3 | Group enr | 0 |
| IP6K3 | inositol hexakisphosphate kinase 3 | 22 | 46 | 39 | 36 | 10 | 31 | 3.2 | Enhanced | 1 |
| C1orf127 | chromosome 1 open reading frame 127 | 5 | 8 | 4 | 12 | 10 | 8 | 3.1 | Group enr | 1 |
| PAX7 | paired box 7 | 3 | 3 | 3 | 3 | 3 | 3 | 3.1 | Enhanced | 0 |
| TRIM7 | tripartite motif containing 7 | 108 | 98 | 105 | 112 | 90 | 102 | 3.1 | Enhanced | 1 |
| ITGB1BP2 | integrin beta 1 binding protein 2 | 15 | 25 | 30 | 25 | 24 | 24 | 3.1 | Group enr | 0 |
| LMO1 | LIM domain only 1 | 14 | 10 | 11 | 15 | 16 | 13 | 3.1 | Group enr | 0 |
| LINGO4 | leucine rich repeat and Ig domain containing 4 | 5 | 8 | 4 | 4 | 4 | 5 | 3.1 | Group enr | 1 |
| PKIA | protein kinase inhibitor alpha | 347 | 496 | 461 | 417 | 402 | 425 | 3.0 | Enhanced | 1 |
| MYO18A | myosin XVIIIA | 395 | 313 | 328 | 414 | 497 | 390 | 3.0 | Group enr | 2 |
| XIRP2 | xin actin-binding repeat containing 2 | 337 | 287 | 365 | 310 | 121 | 284 | 3.0 | Group enr | 1 |
| NRAP | nebulin-related anchoring protein | 787 | 629 | 540 | 713 | 674 | 669 | 3.0 | Group enr | 2 |
| TCEA3 | transcription elongation factor A, 3 | 299 | 280 | 319 | 256 | 356 | 302 | 3.0 | Enhanced | 1 |
| CORO6 | coronin 6 | 194 | 174 | 181 | 193 | 195 | 188 | 3.0 | Enhanced | 1 |
| APOBEC2 | apolipoprotein B mRNA editing enzyme, catalytic polypeptide-like 2 | 333 | 298 | 343 | 349 | 606 | 386 | 3.0 | Group enr | 2 |
| ANKRD2 | ankyrin repeat domain 2 | 66 | 55 | 81 | 257 | 69 | 106 | 2.9 | Group enr | 2 |
| CCDC28B | coiled-coil domain containing 28B | 66 | 59 | 61 | 60 | 76 | 65 | 2.9 | Enhanced | 2 |
| MURC | muscle-related coiled-coil protein | 87 | 134 | 98 | 138 | 84 | 108 | 2.9 | Group enr | 3 |
| HOXC9 | homeobox C9 | 27 | 23 | 14 | 22 | 19 | 21 | 2.9 | Enhanced | 0 |
| PRSS45 | protease, serine, 45 | 2 | 4 | 2 | 4 | 2 | 3 | 2.9 | Enhanced | 1 |
| LMCD1 | LIM and cysteine-rich domains 1 | 117 | 199 | 208 | 185 | 173 | 176 | 2.8 | Enhanced | 1 |
| CSRP3 | cysteine and glycine-rich protein 3 | 259 | 471 | 376 | 567 | 447 | 424 | 2.8 | Group enr | 1 |
| SH3BGR | SH3 domain binding glutamic acid-rich protein | 393 | 243 | 201 | 362 | 275 | 295 | 2.8 | Group enr | 2 |
| RYR3 | ryanodine receptor 3 | 5 | 14 | 16 | 14 | 9 | 12 | 2.8 | Enhanced | 1 |
| AC007421.1 | N/A | 7 | 0 | 0 | 8 | 0 | 3 | 2.8 | Group enr | 0 |
| HHATL | hedgehog acyltransferase-like | 129 | 183 | 154 | 147 | 137 | 150 | 2.7 | Group enr | 1 |
| EEF1A2 | eukaryotic translation elongation factor 1 alpha 2 | 1043 | 792 | 824 | 939 | 1149 | 949 | 2.7 | Enhanced | 4 |
| SYNC | syncoilin, intermediate filament protein | 45 | 36 | 43 | 38 | 43 | 41 | 2.7 | Enhanced | 1 |
| GAMT | guanidinoacetate N-methyltransferase | 363 | 345 | 338 | 346 | 475 | 373 | 2.6 | Group enr | 1 |
| AC037459.4 | Uncharacterized protein | 29 | 24 | 37 | 39 | 29 | 32 | 2.6 | Enhanced | 1 |
| TAL2 | T-cell acute lymphocytic leukemia 2 | 4 | 4 | 5 | 5 | 6 | 5 | 2.6 | Group enr | 1 |
| CAND2 | cullin-associated and neddylation-dissociated 2 | 45 | 40 | 52 | 43 | 60 | 48 | 2.6 | Group enr | 1 |
| PFKM | phosphofructokinase, muscle | 655 | 470 | 546 | 517 | 628 | 563 | 2.6 | Group enr | 1 |
| LSMEM2 | leucine-rich single-pass membrane protein 2 | 7 | 6 | 8 | 6 | 8 | 7 | 2.6 | Group enr | 1 |
| SCN4A | sodium channel, voltage-gated, type IV, alpha subunit | 66 | 44 | 52 | 39 | 67 | 53 | 2.6 | Group enr | 1 |
| FLNC | filamin C, gamma | 279 | 308 | 338 | 369 | 387 | 336 | 2.6 | Enhanced | 1 |
| PPP1R3C | protein phosphatase 1, regulatory subunit 3C | 325 | 454 | 426 | 203 | 265 | 335 | 2.5 | Enhanced | 1 |
| SRPK3 | SRSF protein kinase 3 | 63 | 65 | 68 | 62 | 75 | 67 | 2.5 | Group enr | 1 |
| SBK1 | SH3-binding domain kinase 1 | 15 | 11 | 12 | 17 | 14 | 14 | 2.5 | Group enr | 0 |
| RP11-279O9.4 | Uncharacterized protein | 4 | 2 | 1 | 3 | 2 | 2 | 2.5 | Enhanced | 1 |
| RP11-618P17.4 | N/A | 0 | 2 | 2 | 1 | 0 | 1 | 2.5 | Enhanced | 1 |
| GBX1 | gastrulation brain homeobox 1 | 4 | 12 | 3 | 6 | 8 | 6 | 2.4 | Group enr | 1 |
| FITM1 | fat storage-inducing transmembrane protein 1 | 86 | 125 | 119 | 128 | 137 | 119 | 2.4 | Group enr | 1 |
| TRIM55 | tripartite motif containing 55 | 10 | 17 | 10 | 15 | 9 | 12 | 2.4 | Group enr | 2 |
| KLHL38 | kelch-like family member 38 | 97 | 51 | 70 | 81 | 124 | 85 | 2.4 | Group enr | 2 |
| TMEM110-MUSTN1 | TMEM110-MUSTN1 readthrough | 39 | 13 | 7 | 4 | 28 | 18 | 2.4 | Enhanced | 1 |
| CNKSR1 | connector enhancer of kinase suppressor of Ras 1 | 47 | 57 | 53 | 57 | 63 | 55 | 2.4 | Enhanced | 1 |
| POPDC3 | popeye domain containing 3 | 80 | 84 | 91 | 82 | 116 | 90 | 2.4 | Group enr | 1 |
| CAP2 | CAP, adenylate cyclase-associated protein, 2 | 219 | 193 | 213 | 194 | 212 | 206 | 2.4 | Enhanced | 2 |
| MAMSTR | MEF2 activating motif and SAP domain containing transcriptional regulator | 27 | 33 | 37 | 41 | 29 | 33 | 2.3 | Enhanced | 1 |
| ASB15 | ankyrin repeat and SOCS box containing 15 | 60 | 61 | 36 | 39 | 49 | 49 | 2.3 | Group enr | 1 |
| P2RX6 | purinergic receptor P2X, ligand-gated ion channel, 6 | 23 | 18 | 22 | 17 | 18 | 20 | 2.3 | Enhanced | 2 |
| PGAM2 | phosphoglycerate mutase 2 | 1402 | 1085 | 1175 | 1054 | 1531 | 1250 | 2.3 | Group enr | 2 |
| C11orf89 | chromosome 11 open reading frame 89 | 16 | 10 | 12 | 13 | 20 | 14 | 2.3 | Enhanced | 0 |
| BEST3 | bestrophin 3 | 7 | 20 | 19 | 16 | 13 | 15 | 2.3 | Enhanced | 1 |
| ADPRHL1 | ADP-ribosylhydrolase like 1 | 127 | 90 | 94 | 97 | 151 | 112 | 2.3 | Group enr | 1 |
| RP11-178L8.4 | N/A | 3 | 1 | 4 | 1 | 1 | 2 | 2.2 | Enhanced | 0 |
| MYPN | myopalladin | 80 | 119 | 118 | 113 | 103 | 107 | 2.2 | Group enr | 1 |
| HFE2 | hemochromatosis type 2 | 187 | 221 | 207 | 221 | 262 | 220 | 2.2 | Group enr | 1 |
| HRC | histidine rich calcium binding protein | 174 | 155 | 171 | 152 | 160 | 162 | 2.2 | Group enr | 1 |
| KBTBD12 | kelch repeat and BTB (POZ) domain containing 12 | 24 | 22 | 21 | 22 | 30 | 24 | 2.2 | Group enr | 1 |
| PPAPDC3 | phosphatidic acid phosphatase type 2 domain containing 3 | 68 | 73 | 80 | 72 | 93 | 77 | 2.2 | Group enr | 1 |
| ABLIM2 | actin binding LIM protein family, member 2 | 73 | 50 | 57 | 55 | 79 | 63 | 2.1 | Enhanced | 1 |
| COX6A2 | cytochrome c oxidase subunit VIa polypeptide 2 | 1795 | 1342 | 1274 | 1443 | 1642 | 1499 | 2.1 | Group enr | 0 |
| HSPB7 | heat shock 27kDa protein family, member 7 | 692 | 379 | 640 | 656 | 683 | 610 | 2.1 | Group enr | 1 |
| PTP4A3 | protein tyrosine phosphatase type IVA, member 3 | 266 | 216 | 289 | 237 | 330 | 268 | 2.1 | Group enr | 1 |
| DHRS7C | dehydrogenase/reductase member 7C | 106 | 143 | 140 | 151 | 133 | 135 | 2.1 | Group enr | 2 |
| MYBPH | myosin binding protein H | 0 | 4 | 1 | 5 | 35 | 9 | 2.0 | Group enr | 0 |
| SMCO1 | single-pass membrane protein with coiled-coil domains 1 | 41 | 88 | 62 | 66 | 67 | 65 | 2.0 | Group enr | 1 |
| ALPK2 | alpha-kinase 2 | 5 | 25 | 32 | 16 | 10 | 18 | 2.0 | Enhanced | 4 |
| LMOD3 | leiomodin 3 | 182 | 193 | 191 | 181 | 87 | 167 | 2.0 | Group enr | 1 |
| PEBP4 | phosphatidylethanolamine-binding protein 4 | 257 | 257 | 228 | 273 | 355 | 274 | 2.0 | Enhanced | 1 |
| CECR2 | cat eye syndrome chromosome region, candidate 2 | 12 | 10 | 11 | 12 | 12 | 11 | 2.0 | Enhanced | 1 |
| XIRP1 | xin actin-binding repeat containing 1 | 47 | 51 | 76 | 58 | 91 | 65 | 2.0 | Group enr | 3 |
| TRDN | triadin | 526 | 506 | 498 | 479 | 127 | 427 | 2.0 | Group enr | 1 |
| C15orf27 | chromosome 15 open reading frame 27 | 9 | 6 | 8 | 4 | 10 | 7 | 1.9 | Enhanced | 1 |
| LRRC39 | leucine rich repeat containing 39 | 136 | 221 | 224 | 196 | 120 | 179 | 1.9 | Group enr | 2 |
| SH2B2 | SH2B adaptor protein 2 | 22 | 16 | 24 | 18 | 32 | 23 | 1.9 | Enhanced | 1 |
| PPP1R3A | protein phosphatase 1, regulatory subunit 3A | 79 | 74 | 73 | 68 | 20 | 63 | 1.9 | Group enr | 2 |
| SGCA | sarcoglycan, alpha | 200 | 182 | 197 | 171 | 183 | 186 | 1.9 | Group enr | 1 |
| RP11-131H24.4 | Uncharacterized protein | 3 | 2 | 1 | 2 | 3 | 2 | 1.9 | Enhanced | 0 |
| SPEG | SPEG complex locus | 94 | 75 | 109 | 93 | 111 | 96 | 1.9 | Enhanced | 1 |
| JPH2 | junctophilin 2 | 71 | 59 | 71 | 79 | 88 | 74 | 1.9 | Enhanced | 1 |
| CA14 | carbonic anhydrase XIV | 7 | 14 | 24 | 15 | 11 | 14 | 1.8 | Enhanced | 1 |
| MYOM2 | myomesin 2 | 193 | 200 | 260 | 230 | 254 | 227 | 1.8 | Group enr | 2 |
| CLTCL1 | clathrin, heavy chain-like 1 | 30 | 26 | 26 | 30 | 38 | 30 | 1.8 | Enhanced | 0 |
| NMRK2 | nicotinamide riboside kinase 2 | 202 | 134 | 167 | 216 | 232 | 190 | 1.8 | Group enr | 1 |
| EYA4 | eyes absent homolog 4 | 10 | 12 | 13 | 7 | 7 | 10 | 1.8 | Enhanced | 3 |
| PTGES3L | prostaglandin E synthase 3 (cytosolic)-like | 60 | 75 | 70 | 57 | 79 | 68 | 1.8 | Group enr | 0 |
| TXLNB | taxilin beta | 88 | 149 | 110 | 119 | 87 | 111 | 1.8 | Group enr | 3 |
| SIX1 | SIX homeobox 1 | 40 | 62 | 65 | 78 | 81 | 65 | 1.8 | Group enr | 2 |
| PPP2R3A | protein phosphatase 2, regulatory subunit B'', alpha | 62 | 92 | 77 | 86 | 52 | 74 | 1.8 | Enhanced | 2 |
| SYNPO | synaptopodin | 418 | 377 | 378 | 402 | 559 | 427 | 1.8 | Enhanced | 3 |
| S100A1 | S100 calcium binding protein A1 | 1135 | 840 | 496 | 859 | 1058 | 877 | 1.7 | Group enr | 2 |
| COX7A1 | cytochrome c oxidase subunit VIIa polypeptide 1 | 687 | 775 | 768 | 895 | 1013 | 828 | 1.7 | Group enr | 0 |
| LRRC2 | leucine rich repeat containing 2 | 45 | 87 | 63 | 72 | 52 | 64 | 1.7 | Group enr | 2 |
| NANOS1 | nanos homolog 1 | 23 | 7 | 19 | 5 | 18 | 14 | 1.7 | Enhanced | 1 |
| SYNPO2L | synaptopodin 2-like | 119 | 96 | 90 | 109 | 119 | 107 | 1.7 | Group enr | 2 |
| TCAP | titin-cap | 2252 | 2543 | 2619 | 3053 | 3132 | 2720 | 1.7 | Group enr | 2 |
| PACSIN3 | protein kinase C and casein kinase substrate in neurons 3 | 149 | 125 | 207 | 160 | 168 | 162 | 1.7 | Enhanced | 2 |
| KLHL31 | kelch-like family member 31 | 52 | 61 | 57 | 61 | 55 | 57 | 1.7 | Enhanced | 1 |
| CTC-260F20.3 | Uncharacterized protein; YjeF N-terminal domain-containing protein 3 | 51 | 121 | 129 | 126 | 134 | 112 | 1.7 | Enhanced | 0 |
| CLIC5 | chloride intracellular channel 5 | 147 | 145 | 134 | 110 | 109 | 129 | 1.6 | Enhanced | 0 |
| TMEM52 | transmembrane protein 52 | 109 | 151 | 157 | 172 | 121 | 142 | 1.6 | Group enr | 1 |
| COQ10A | coenzyme Q10 homolog A | 177 | 158 | 159 | 145 | 133 | 154 | 1.6 | Group enr | 0 |
| SGCG | sarcoglycan, gamma | 43 | 77 | 59 | 64 | 80 | 64 | 1.6 | Enhanced | 2 |
| LDB3 | LIM domain binding 3 | 807 | 853 | 1025 | 992 | 1003 | 936 | 1.6 | Group enr | 1 |
| FSD2 | fibronectin type III and SPRY domain containing 2 | 49 | 60 | 46 | 57 | 59 | 54 | 1.6 | Group enr | 1 |
| PTGES3L-AARSD1 | PTGES3L-AARSD1 readthrough | 3 | 7 | 7 | 3 | 1 | 4 | 1.6 | Enhanced | 3 |
| RP11-178C3.1 | Uncharacterized protein | 0 | 4 | 4 | 0 | 2 | 2 | 1.6 | Enhanced | 0 |
| PRPH2 | peripherin 2 | 5 | 4 | 4 | 5 | 8 | 5 | 1.6 | Enhanced | 1 |
| SYNPO2 | synaptopodin 2 | 276 | 281 | 332 | 304 | 332 | 305 | 1.5 | Enhanced | 1 |
| LMOD2 | leiomodin 2 | 251 | 320 | 296 | 395 | 342 | 321 | 1.5 | Group enr | 1 |
| RBM24 | RNA binding motif protein 24 | 192 | 157 | 188 | 175 | 169 | 176 | 1.5 | Enhanced | 0 |
| SMYD1 | SET and MYND domain containing 1 | 146 | 176 | 184 | 167 | 195 | 173 | 1.5 | Group enr | 1 |
| TBX15 | T-box 15 | 45 | 44 | 47 | 47 | 48 | 46 | 1.5 | Group enr | 1 |
| ATP1A2 | ATPase, Na+/K+ transporting, alpha 2 polypeptide | 413 | 332 | 351 | 354 | 297 | 349 | 1.5 | Group enr | 1 |
| SPTB | spectrin, beta, erythrocytic | 47 | 64 | 60 | 52 | 60 | 57 | 1.5 | Group enr | 4 |
| PADI2 | peptidyl arginine deiminase, type II | 110 | 85 | 114 | 136 | 113 | 112 | 1.5 | Enhanced | 1 |
| CKMT2 | creatine kinase, mitochondrial 2 | 415 | 555 | 589 | 496 | 504 | 512 | 1.5 | Group enr | 1 |
| DENND2C | DENN/MADD domain containing 2C | 30 | 35 | 22 | 32 | 37 | 31 | 1.5 | Group enr | 1 |
| ASB11 | ankyrin repeat and SOCS box containing 11 | 54 | 62 | 41 | 47 | 67 | 54 | 1.5 | Group enr | 1 |
| TUBA8 | tubulin, alpha 8 | 138 | 118 | 144 | 121 | 141 | 132 | 1.5 | Group enr | 3 |
| DNAJB5 | DnaJ homolog, subfamily B, member 5 | 71 | 80 | 71 | 77 | 65 | 73 | 1.5 | Enhanced | 1 |
| TPM1 | tropomyosin 1 | 4153 | 2663 | 3257 | 3372 | 3740 | 3437 | 1.4 | Group enr | 3 |
| GPR157 | G protein-coupled receptor 157 | 40 | 14 | 19 | 36 | 49 | 31 | 1.4 | Enhanced | 1 |
| TMEM182 | transmembrane protein 182 | 49 | 74 | 62 | 63 | 59 | 61 | 1.4 | Group enr | 1 |
| ALPK3 | alpha-kinase 3 | 66 | 54 | 66 | 80 | 77 | 69 | 1.4 | Group enr | 1 |
| SLC2A4 | solute carrier family 2, member 4 | 85 | 88 | 97 | 89 | 86 | 89 | 1.4 | Enhanced | 1 |
| ANO5 | anoctamin 5 | 28 | 30 | 39 | 24 | 16 | 27 | 1.4 | Enhanced | 1 |
| DCUN1D2 | DCN1, defective in cullin neddylation 1, domain containing 2 | 70 | 64 | 87 | 79 | 77 | 75 | 1.4 | Group enr | 1 |
| SLC25A4 | solute carrier family 25, member 4 | 893 | 958 | 909 | 895 | 870 | 905 | 1.4 | Group enr | 1 |
| PFKFB1 | 6-phosphofructo-2-kinase/fructose-2,6-biphosphatase 1 | 38 | 38 | 36 | 42 | 30 | 37 | 1.4 | Group enr | 0 |
| KCNS3 | potassium voltage-gated channel, delayed-rectifier, subfamily S, member 3 | 34 | 33 | 25 | 37 | 46 | 35 | 1.4 | Enhanced | 1 |
| PRSS42 | protease, serine, 42 | 1 | 4 | 3 | 7 | 1 | 3 | 1.3 | Group enr | 1 |
| AL138781.1 | CDNA FLJ26691 fis, clone MPG07564; Uncharacterized protein | 2 | 1 | 1 | 1 | 1 | 1 | 1.3 | Enhanced | 0 |
| CTD-2207O23.12 | Uncharacterized protein | 0 | 18 | 15 | 0 | 1 | 7 | 1.3 | Enhanced | 0 |
| NEXN | nexilin | 290 | 354 | 332 | 236 | 48 | 252 | 1.3 | Enhanced | 1 |
| HOXC10 | homeobox C10 | 60 | 44 | 50 | 51 | 51 | 51 | 1.3 | Enhanced | 2 |
| SMPX | small muscle protein, X-linked | 306 | 416 | 360 | 422 | 413 | 383 | 1.3 | Group enr | 0 |
| PDE4DIP | phosphodiesterase 4D interacting protein | 1358 | 1657 | 1449 | 1441 | 1346 | 1450 | 1.3 | Group enr | 2 |
| CTD-2210P24.4 | Uncharacterized protein | 4 | 3 | 3 | 2 | 7 | 4 | 1.3 | Enhanced | 0 |
| OR10AB1P | olfactory receptor, family 10, subfamily AB, member 1 pseudogene | 2 | 1 | 1 | 1 | 1 | 1 | 1.3 | Enhanced | 1 |
| AGBL1 | ATP/GTP binding protein-like 1 | 2 | 2 | 1 | 4 | 3 | 2 | 1.3 | Enhanced | 1 |
| MYH7B | myosin, heavy chain 7B, cardiac muscle, beta | 36 | 81 | 70 | 59 | 29 | 55 | 1.2 | Group enr | 0 |
| TNNC1 | troponin C type 1 | 2647 | 4172 | 4115 | 4012 | 3627 | 3715 | 1.2 | Group enr | 2 |
| BVES | blood vessel epicardial substance | 17 | 18 | 21 | 20 | 18 | 19 | 1.2 | Enhanced | 2 |
| MYH7 | myosin, heavy chain 7, cardiac muscle, beta | 1415 | 2554 | 2682 | 2066 | 2040 | 2151 | 1.2 | Group enr | 3 |
| TRIM63 | tripartite motif containing 63, E3 ubiquitin protein ligase | 149 | 123 | 80 | 145 | 193 | 138 | 1.2 | Group enr | 0 |
| MYOM3 | myomesin 3 | 78 | 88 | 111 | 79 | 129 | 97 | 1.2 | Group enr | 2 |
| HSPB6 | heat shock protein, alpha-crystallin-related, B6 | 696 | 612 | 488 | 796 | 732 | 665 | 1.2 | Enhanced | 3 |
| MYL2 | myosin, light chain 2, regulatory, cardiac, slow | 3617 | 4706 | 5194 | 5125 | 4481 | 4625 | 1.1 | Group enr | 2 |
| CDNF | cerebral dopamine neurotrophic factor | 20 | 20 | 24 | 27 | 20 | 22 | 1.1 | Enhanced | 1 |
| ACTN2 | actinin, alpha 2 | 1141 | 1010 | 1100 | 1218 | 1149 | 1124 | 1.1 | Group enr | 2 |
| FZD9 | frizzled family receptor 9 | 1 | 1 | 1 | 4 | 2 | 2 | 1.1 | Enhanced | 0 |
| TMX2-CTNND1 | TMX2-CTNND1 readthrough | 4 | 0 | 0 | 2 | 0 | 1 | 1.1 | Enhanced | 0 |
| MYO18B | myosin XVIIIB | 59 | 58 | 61 | 79 | 91 | 70 | 1.1 | Group enr | 1 |
| ASB18 | ankyrin repeat and SOCS box containing 18 | 6 | 6 | 3 | 6 | 4 | 5 | 1.1 | Group enr | 0 |
| DOK5 | docking protein 5 | 26 | 13 | 20 | 17 | 25 | 20 | 1.1 | Enhanced | 1 |
| ADCY2 | adenylate cyclase 2 | 74 | 71 | 77 | 76 | 92 | 78 | 1.1 | Enhanced | 3 |
| FBXO40 | F-box protein 40 | 52 | 69 | 63 | 56 | 73 | 63 | 1.1 | Group enr | 0 |
| SRL | sarcalumenin | 164 | 215 | 243 | 180 | 229 | 206 | 1.1 | Group enr | 2 |
| DUSP27 | dual specificity phosphatase 27 | 43 | 39 | 42 | 41 | 58 | 44 | 1.1 | Group enr | 1 |
| P2RY2 | purinergic receptor P2Y, G-protein coupled, 2 | 16 | 13 | 21 | 13 | 16 | 16 | 1.1 | Enhanced | 1 |
| HSPB2 | Homo sapiens heat shock 27kDa protein 2, mRNA. | 69 | 58 | 63 | 74 | 86 | 70 | 1.0 | Enhanced | 2 |
| PPP1R1A | protein phosphatase 1, regulatory subunit 1A | 196 | 233 | 262 | 266 | 249 | 241 | 1.0 | Enhanced | 2 |
| DDN | dendrin | 14 | 11 | 17 | 23 | 172 | 48 | 1.0 | Group enr | 1 |
| MYL3 | myosin, light chain 3, alkali; ventricular, skeletal, slow | 790 | 1517 | 1346 | 1161 | 1384 | 1239 | 1.0 | Group enr | 3 |
| USP2 | ubiquitin specific peptidase 2 | 50 | 84 | 67 | 71 | 61 | 67 | 1.0 | Enhanced | 2 |
| MYOM1 | myomesin 1 | 171 | 334 | 343 | 280 | 263 | 278 | 1.0 | Group enr | 2 |
| UNC45B | unc-45 homolog B | 73 | 70 | 74 | 65 | 91 | 75 | 1.0 | Group enr | 1 |
| C1QTNF9 | C1q and tumor necrosis factor related protein 9 | 2 | 4 | 5 | 3 | 3 | 3 | 1.0 | Enhanced | 1 |
| AL354898.1 | N/A | 2 | 1 | 1 | 1 | 1 | 1 | 1.0 | Enhanced | 0 |
| CARNS1 | carnosine synthase 1 | 54 | 39 | 32 | 43 | 33 | 40 | 1.0 | Group enr | 2 |
| TMEM233 | transmembrane protein 233 | 16 | 12 | 9 | 15 | 18 | 14 | 1.0 | Group enr | 1 |
| KY | kyphoscoliosis peptidase | 13 | 5 | 3 | 8 | 5 | 7 | 1.0 | Enhanced | 1 |
| SIX4 | SIX homeobox 4 | 5 | 6 | 6 | 6 | 7 | 6 | 1.0 | Enhanced | 1 |
| IL17D | interleukin 17D | 19 | 69 | 54 | 56 | 63 | 53 | 1.0 | Group enr | 1 |
| ART3 | ADP-ribosyltransferase 3 | 105 | 117 | 122 | 88 | 70 | 100 | 1.0 | Group enr | 1 |
| RP11-599B13.6 | Uncharacterized protein | 0 | 6 | 4 | 0 | 4 | 3 | 0.9 | Enhanced | 0 |
| NPHP1 | nephronophthisis 1 | 36 | 24 | 26 | 18 | 16 | 24 | 0.9 | Enhanced | 1 |
| KCNT1 | potassium channel, subfamily T, member 1 | 8 | 7 | 12 | 9 | 11 | 9 | 0.9 | Enhanced | 1 |
| PPM1J | protein phosphatase, Mg2+/Mn2+ dependent, 1J | 29 | 39 | 40 | 37 | 18 | 33 | 0.9 | Group enr | 1 |
| NKAIN1 | Na+/K+ transporting ATPase interacting 1 | 9 | 7 | 9 | 9 | 10 | 9 | 0.9 | Group enr | 1 |
| LRRC38 | leucine rich repeat containing 38 | 17 | 16 | 17 | 4 | 10 | 13 | 0.9 | Group enr | 1 |
| EXTL1 | exostosin-like glycosyltransferase 1 | 5 | 5 | 6 | 6 | 7 | 6 | 0.8 | Enhanced | 1 |
| UPK3A | uroplakin 3A | 6 | 22 | 6 | 22 | 5 | 12 | 0.8 | Group enr | 2 |
| SLC8A3 | solute carrier family 8, member 3 | 4 | 9 | 10 | 7 | 9 | 8 | 0.8 | Group enr | 1 |
| USP6 | ubiquitin specific peptidase 6 | 4 | 21 | 7 | 10 | 4 | 9 | 0.7 | Group enr | 1 |
| RBFOX1 | RNA binding protein, fox-1 homolog 1 | 43 | 51 | 43 | 38 | 25 | 40 | 0.7 | Group enr | 1 |
| KCNQ5 | potassium voltage-gated channel, KQT-like subfamily, member 5 | 8 | 12 | 11 | 11 | 4 | 9 | 0.7 | Enhanced | 1 |
| C20orf26 | chromosome 20 open reading frame 26 | 8 | 3 | 13 | 8 | 6 | 7 | 0.7 | Group enr | 0 |
| TCF15 | transcription factor 15 | 2 | 5 | 5 | 5 | 7 | 5 | 0.7 | Group enr | 1 |
| PAX3 | paired box 3 | 3 | 1 | 1 | 2 | 3 | 2 | 0.7 | Enhanced | 1 |
| TTC40 | tetratricopeptide repeat domain 40 | 8 | 7 | 7 | 5 | 12 | 8 | 0.7 | Enhanced | 0 |
| HRASLS | HRAS-like suppressor | 32 | 33 | 30 | 32 | 32 | 32 | 0.7 | Group enr | 1 |
| RP11-426L16.10 | Rho-related GTP-binding protein RhoC | 2 | 6 | 3 | 1 | 2 | 3 | 0.6 | Enhanced | 0 |
| SLC36A2 | solute carrier family 36, member 2 | 47 | 49 | 43 | 29 | 26 | 39 | 0.6 | Group enr | 1 |
| MAP6D1 | MAP6 domain containing 1 | 10 | 14 | 19 | 11 | 18 | 14 | 0.6 | Group enr | 1 |
| GAS2L2 | growth arrest-specific 2 like 2 | 1 | 0 | 1 | 0 | 3 | 1 | 0.6 | Enhanced | 1 |
| SIX2 | SIX homeobox 2 | 10 | 6 | 10 | 8 | 6 | 8 | 0.6 | Enhanced | 1 |
| CAMK2A | calcium/calmodulin-dependent protein kinase II alpha | 61 | 62 | 65 | 72 | 85 | 69 | 0.5 | Group enr | 4 |
| SAMD4A | sterile alpha motif domain containing 4A | 78 | 51 | 63 | 55 | 72 | 64 | 0.5 | Group enr | 1 |
| AL109927.1 | HCG2032222; PRO2047; Uncharacterized protein | 14 | 0 | 0 | 0 | 0 | 3 | 0.5 | Enhanced | 0 |
| ATP2B2 | ATPase, Ca++ transporting, plasma membrane 2 | 24 | 17 | 15 | 18 | 27 | 20 | 0.5 | Enhanced | 1 |
| CAMK2B | calcium/calmodulin-dependent protein kinase II beta | 44 | 39 | 41 | 45 | 52 | 44 | 0.5 | Group enr | 5 |
| PITX2 | paired-like homeodomain 2 | 21 | 35 | 32 | 31 | 27 | 29 | 0.5 | Enhanced | 0 |
| ST8SIA5 | ST8 alpha-N-acetyl-neuraminide alpha-2,8-sialyltransferase 5 | 4 | 2 | 4 | 2 | 4 | 3 | 0.5 | Group enr | 2 |
| MLF1 | myeloid leukemia factor 1 | 108 | 68 | 93 | 58 | 131 | 92 | 0.4 | Group enr | 1 |
| AC011239.1 | Uncharacterized protein | 2 | 0 | 2 | 2 | 3 | 2 | 0.4 | Group enr | 0 |
| ARPP21 | cAMP-regulated phosphoprotein, 21kDa | 18 | 22 | 29 | 27 | 17 | 22 | 0.4 | Group enr | 1 |
| ASB4 | ankyrin repeat and SOCS box containing 4 | 9 | 17 | 12 | 15 | 17 | 14 | 0.4 | Group enr | 1 |
| MYH8 | myosin, heavy chain 8, skeletal muscle, perinatal | 1 | 6 | 2 | 1 | 18 | 6 | 0.4 | Enhanced | 2 |
| C1orf167 | chromosome 1 open reading frame 167 | 1 | 12 | 11 | 15 | 8 | 10 | 0.3 | Group enr | 1 |
| ART5 | ADP-ribosyltransferase 5 | 3 | 4 | 5 | 5 | 5 | 4 | 0.3 | Group enr | 2 |
| FAM166B | family with sequence similarity 166, member B | 90 | 132 | 110 | 146 | 121 | 120 | 0.3 | Group enr | 1 |
| AL158147.2 | HCG2011465; Uncharacterized protein | 0 | 0 | 0 | 17 | 0 | 3 | 0.3 | Group enr | 0 |
| ARX | aristaless related homeobox | 7 | 7 | 6 | 7 | 9 | 7 | 0.3 | Group enr | 0 |
| GSDMC | gasdermin C | 5 | 12 | 18 | 21 | 2 | 12 | 0.3 | Group enr | 1 |
| PVALB | parvalbumin | 12 | 0 | 1 | 1 | 36 | 10 | 0.2 | Group enr | 2 |
| SIM2 | single-minded homolog 2 | 5 | 4 | 6 | 3 | 5 | 4 | 0.2 | Group enr | 1 |
| GHR | growth hormone receptor | 16 | 26 | 21 | 20 | 19 | 20 | 0.2 | Group enr | 0 |

^1^ RNA category of skeletal muscle elevated expression defined as enriched, group enriched (group enr) or enhanced.
